# Supplementary material for: Cell migration guided by long-lived spatial memory
Source: Nat Commun. 2021 Jul 5;12:4118. doi: 10.1038/s41467-021-24249-8 (PMC8257581; doi:10.1038/s41467-021-24249-8)
Supplement: Supplementary file 1 — Supplementary Information [file 41467_2021_24249_MOESM1_ESM.pdf]

Cell migration guided by long-lived spatial memory
Supplementary material

Joseph d'Alessandro,[1,*] Alex Barbier-Chebbah,[2] Victor Cellerin,[1] Olivier Benichou,[2] René-Marc Mège,[1] Raphaël Voituriez,[2,3,†] and Benoit Ladoux[1,‡]

[1]*Institut Jacques Monod, CNRS UMR 7592, University Paris Diderot, Paris 75013, France*
[2]*Laboratoire de Physique Théorique de la Matière Condensée, UMR 7600 CNRS, Sorbonne Université, 75005 Paris, France*
[3]*Laboratoire Jean Perrin, UMR 8237 CNRS, Sorbonne Université, 75005 Paris, France*

I. Supplementary information – experiments

The Supplementary Figures 1 shows a substantial subset of the experimental cell trajectories on tracks of three different widths $W = 10, 20, 50\,\mu\mathrm{m}$ (here, only trajectories of $> 40\,\mathrm{h}$ have been retained). From those plots, one can appreciate at the same time both the generic aspect of the oscillation phenomenon and its variability. In what follows we tackle various aspects of this phenomenon.

A. Cell length and polarisation

By comparing the cell length in the two stereotypical examples shown in Figure 1 (static and oscillating cell), it appears that they share the same long time spreading dynamics – which might be largely due to mitomycin C treatment. The difference in the two behaviours should then arise simply from the capacity of cells to polarise strongly or not. Supplementary Figure 1e-f shows that an oscillating cell is able to repolarise completely during reversals and hence to migrate very persistently between the two ends of the footprint.

B. Oscillation analysis

Despite the wide variability in cellular behaviours, conserved patterns seem to emerge from the different cell trajectories. Therefore we resorted to a semi-manual analysis of the oscillations. Oscillation peaks were first automatically detected on the centre-of-mass trajectory, using an algorithm based on the Mexican hat wavelet transform. Then the output was manually corrected so as to remove false positive (mostly small amplitude undulations in zones with little movement). For each peak, the corresponding proximal extremum of the cell edge position was then searched, so that the final peak position corresponded to the actual extremal point reached by the cell edge, irrespective of the cell length that could vary a lot especially during reversals. Then we analysed the time series of the amplitude and period of the so-called "hemi-periods" (one peak-to-peak run, corresponding to half of an oscillation). We noticed that most of the time a robust linear relation $A \propto T$ emerges, pointing to constant speed motion despite the long-term variation of oscillation amplitude. The time series of amplitudes were also consistently increasing with time, suggesting the existence of an extending accessible domain. Moreover, despite consistent change of slope at $A \simeq 70 - 100\,\mu\mathrm{m}$, the (A_i) sequences grew roughly linearly, which is consistent with the idea of the addition of a constant "discovered" area at each iteration. Finally, it is interesting to note that in spite of differences in the full time cell trajectories, the oscillations could share their properties locally. It is well examplified in Supplementary Figure 2 where $A(T)$ and (A_i) plots locally super-impose when the oscillations are in the same dynamical range – $t \in [30; 50]\,\mathrm{h}$ for cell 1, $t \in [10; 30]\,\mathrm{h}$ for cell 2 – while the two cells' history before and after differ completely.

C. The phase-space of linear cell migration.

Here we sought to extend the approach taken in the work by Bruckner *et al.* [1] for a case in which space is not bounded but footprint effects are taken into account. The idea is to draw a generic description of the cell behaviour by analysing the motion through the measurement of average and standard deviation of acceleration at any point in a proper phase space. The main task in that context is to replace the space variable – in unbounded space, x-invariance should be warranted – by suitable footprint variables. Both the speed and $\Delta\varphi$, the difference in footprint value between the two cell ends, showed significant correlations with the cell acceleration. To get a better estimate of the relation between $\Delta\varphi$ and a, we represented a as function of both $\Delta\varphi$ and φ_c, the value at the cell centre. It appeared that $\langle a\rangle$ took significant values mostly at $\|\Delta\varphi\| = \varphi_c$, with an acceleration oriented towards higher φ values (*ie* towards the interior of the footprint). This is shown in Supplementary Figure 3–4, with smoothed heatmaps highlighting the nonzero values of $\langle a\rangle$. This suggested that the footprint had an effect on the cell motion primarily

* Electronic address: joseph.dalessandro@ijm.fr
† Electronic address: voiturie@lptmc.jussieu.fr
‡ Electronic address: benoit.ladoux@ijm.fr

near the footprint edges ($\|\Delta\varphi\| \simeq \varphi_c$ implies that $\varphi \simeq 0$ on one of the cell ends). To investigate this further, we decided to represent $\langle a\rangle$ as a function of v, φ_l and φ_r (Supplementary Figure 5). This made clear that the main effect of the footprint was restricted to the small zones where $\varphi_l \leqslant \varphi_0$ or $\varphi_r \leqslant \varphi_0$, with a critical threshold value $\varphi_0 \simeq 5\,\mathrm{h}$. Following this observation, we distinguished between the interior of the footprint and its borders based on this criterium on $\varphi_{l,r}$ and studied further the relationship between a and v in the two zones. Within the footprint, the data seem to follow a linear relationship, $\langle a\rangle = v/\tau_{va}$, which is common in persistent random cell migration. On the edge of the footprint, $\langle a\rangle$ is oriented inwards irrespective of the sign of v with a parabolic relation between the two variables.

To verify that the $a - \varphi$ relationship is not an artifact of the overall increase of φ with time, we reproduced the same measurements on limited time windows. We found that the output does not depend on the time of measurement, confirming that the observed effects are real effects of v and φ. Finally, the time traces of v, φ_l and φ_r in Supplementary Figure 6 illustrate the motion of two stereotypical cells in this phase space.

D. Analysis of experimental trajectories through the PSAW model.

We discretised the trajectories to focus on the statistics of motion reversals independently from the detailed dynamics of cell speed and shape fluctuations. The main issue was to determine the right space and time scales to define the discretisation grids. In the framework of the PSAW model, the most natural spatial scale is the cell size, but as seen in Supplementary Figure 1, it exhibits both slow growth and fast fluctuations. As a simpler proxy, we used the minimum of the cell size in time as a reference length, $L_{\mathrm{ref}} = \min_t L$, to define the spatial grid (Supplementary Figure 7b). With that convention and the time resolution of our experiments $\Delta t = 6\,\mathrm{min}$, at each time frame the cells either stay on the same site or jump to a neighbouring site. We redefined the time grid by considering only the jump events (Supplementary Figure 7c), ignoring that jump times vary, as shown by their distribution (Supplementary Figure 7d), with an average of $\langle t_j\rangle = 0.6\,\mathrm{h}$. As a self-consistency check, we simulated PSAWs in the range of parameters found experimentally, then we expanded time by randomly picking jump times in the experimental t_j distribution and smoothed the trajectories in space using a sliding average over $2\,\mathrm{h}$. Then we applied the discretisation procedure to those artificial trajectories and checked that the output (k, β) couples are consistent with the input ones. This procedure is also robust to the choice of L_{ref} as shown in Supplementary Figure f by the very slight variation of the ouptut k distribution when the median of $L(t)$ or a single L_{ref} value for all cells was used instead of the minimum.

We found that the experimental (k, β) points are concentrated around the $\beta = -2(k+1)$ line irrespective of the track width. Since it means that the reversal probability on the footprint edge is constant, it suggests that the main difference between oscillating and static cells resides in their ability to polarise persistently rather than in the self-attraction strength of their trajectory. Interestingly, the k distribution is slightly shifted for $W = 10\,\mu\mathrm{m}$ which also displays less oscillating cells compared to the larger tracks. On conditioned substrates on the other hand, the (k, β) scatter is moved to higher β values, with a non-negligible fraction of positive β, which evidences a reduced effect of the cell path's self-attraction in that condition.

II. Supplementary information - model

This Supplementary Material presents definitions of the random walk models discussed in the main text, and summarizes their main properties. We discuss

- the class of attractive self interacting random walks
- the SATW and PSATW processes for $d = 1$
- the SATW process for $d > 1$

A. Attractive self interacting random walks

In this subsection we give the definitions of the self interacting random walks of the attractive class discussed in the main text, and summarize their important properties.

1. *definitions*

Self interacting random walks can be defined as nearest neighbor random walks on a d-dimensional lattice, for which the probability to jump to a neighboring site i at time t is proportional to a weight function $w(n_i)$ that depends on the number of previous visits n_i of the random walker to site i up to time t. Note that alternatively the variable n can be defined on bonds instead of sites ; this prescription will be called bond convention below, and will not be discussed in details. Denoting by x_t the position of the random walker at time t and by $V(x_t)$ the set of neighboring sites, the transition probability defining the process

can be written for $k \in V(x_t)$:

$$p(x_{t+1} = k|\{x_t, ..., x_0\}) = \frac{w(n_k)}{\sum_{j \in V(x_t)} w(n_j)}. \tag{S1}$$

Note that the set of local times (or number of visits) $\{n_i\}_{i \in \mathbb{Z}^d}$ at time t depends on the full trajectory $\{x_t, ..., x_0\}$ up to time t. The process is therefore strongly non Makovian and has long range memory. We focus in this paper on the attractive case, which is realized by weight functions w that are monotonically increasing with n. Previous works have investigated exponential $[w(n) = e^{-\beta n}, \beta < 0]$, subexponential $[w(n) = e^{-\beta n^k}, \beta < 0]$, polynomial $[w(n) = n^\beta, \beta > 0]$ or asymptotically constant $[w(n) \sim 1]$ weight functions[2–5] ; some of the properties of these processes are reminded below.

2. *self-trapping*

Qualitatively, attractive self interacting random walks are attracted by their own path. Strikingly, this can lead to the full trapping of the walker within a finite set of sites in the $t \to \infty$ limit, and therefore to a bounded mean squared displacement (MSD). This effect was demonstrated mathematically by B. Davis[2] for 1–dimensional attractive self interacting random walks with the bond convention. This result states that if $\sum_{n=1}^{\infty} w(n)^{-1} = \infty$ the random walker is free and will visit infinitely often all the sites of the lattice. Conversely, for $\sum_{n=1}^{\infty} w(n)^{-1} < \infty$, the random walker visits only a finite set of sites and will eventually almost surely oscillate between two adjacent sites. Even if no mathematical proof is available, these results are conjectured to apply more generally to the site convention that we use in this paper, and for d–dimensional lattices [6]. Finally, this indicates that exponential or subexponantial or polynomial (with $\beta > 1$) attractive self interacting random walks lead to the full trapping of the random walker and to a bounded MSD. We focus below on the class of free attractive self interacting random walks, for which the MSD diverges for $t \to \infty$.

3. *free attractive self interacting random walks*

In this subsection we provide a brief survey of attractive self interacting random walks that are free (ie have diverging MSD for $t \to \infty$), before focusing on the models that are used in paper (SATW and PSATW).

a. Polynomial self interacting random walks

This model is defined by the transition probability (S1) with the weight function[3]:

$$w(n) = \frac{1}{|1-\alpha|}(n/2)^\alpha - \frac{B}{(1-\alpha)^2}(n/2)^{\alpha-1} + O(n^{\alpha-2}) \text{ where } B \in \mathbb{R}. \tag{S2}$$

So far it has been studied mostly for $d = 1$. The Davis theorem stated above and its generalization show that for $1 < \alpha$ the random walker is fully trapped. For $0 < \alpha < 1$ the walk is free (the MSD diverges for $t \to \infty$), and the scaling of the MSD has been determined in $1d$ for the bond convention : $\langle x_t^2 \rangle \sim t^{\frac{2(1-\alpha)}{2-\alpha}}$. Note that numerical results indicate that this scaling does not always hold for the site convention. The persistence exponent is not known for this process, as well as the scaling of the MSD for $d > 1$.

b. Self interacting random walks with bounded weights : the self attracting walk (SATW)

This model is defined by the transition probability (S1) with the weight function [7–9]:

$$w(n) = \exp(-\beta f(n)), \tag{S3}$$

where $f(0) = 0$ and $f(n > 0) = 1$ and $\beta < 0$. In this regime, for $d = 1$, the random walk is free and the scaling of the MSD is diffusive[10] : $\langle x_t^2 \rangle \sim t$. This model, with its generalization to persistent random walks discussed below, is the central model on which we focus in this paper. This choice more generally covers all cases where f is bounded. For practical applications, and in particular in the context of cell migration that we study in this paper, it means that either the deposited signal saturates, or the cell response to the deposited signal saturates. While such hypothesis –even if realistic– cannot be directly challenged experimentally, our experimental results are consistent with this model. Of note, the SATW dynamics at time t is fully determined by the position of the walker x_t and the visited territory $\mathcal{D}_t$, ie the set of sites that have been visited up to time t. For $d = 1$, $\mathcal{D}_t$ is a connected segment, which greatly simplifies the analysis as compared to $d > 1$. When the walker is inside $\mathcal{D}_t$ (away from its boundaries) the dynamics is that of a symmetric Polya walk ; the dynamics is modified only when the walker reaches the boundary of $\mathcal{D}_t$. The properties of the SATW model will be discussed in more details in the next subsections.

c. The persistent self attracting walk (PSATW)

To take into account the persistence of migrating cells, we make use in this paper of a generalization of the SATW called persistent self attracting walk (PSATW) [11]. For $d = 1$, it is defined as follows. When the walker is on a site i within the visited domain $\mathcal{D}_t$ – ie surrounded by sites that have already been visited, $n_{i-1}, n_{i+1} > 0$ – it performs a classical persistent random random walk : it changes direction with probability $p_{r,i} = \frac{e^{-k}}{e^{-k}+e^{k}}$, and reproduces its previous step with probability $1 - p_{r,i}$. Here $k > 0$ is a parameter that controls the cell persistence length $l_p = e^{2k}$, or equivalently persistence time $t_p = l_p$ (the speed is set to 1 in this discrete model). When the walker is at an edge of the domain $\mathcal{D}_t$ (eg $n_{i+1} > 0$), it experiences a local bias inward the visited domain parametrized by $\beta < 0$ and the probability to change direction can be written $p_{r,e} = \frac{e^{-k-\beta}}{e^{-k-\beta}+e^{k}}$, while the probability to reproduce the previous step is $1 - p_{r,e}$. In particular for $k = 0$ the SATW model is recovered ; for $\beta = 0$ the classical persistent random walk is recovered. For $d = 1$, the random walk is free and the scaling of the MSD is diffusive in the long time limit [11] : $\langle x_t^2 \rangle \sim t$. The properties of the PSATW model will be discussed in more details in the next subsections.

d. Asymptotically free walk :

This model is defined by the transition probability (S1) with the weight function [7–9] :

$$w(n) = 1 - 2Bn^{-1} + O(n^{-2}) \text{ where } B \in \mathbb{R}. \tag{S4}$$

It was introduced as a refinement of the SATW ; for $d = 1$, the MSD is diffusive as for the SATW. The persistence exponent is not known for this process, as well as its properties for $d > 1$.

B. SATW and PSATW for $d = 1$.

In this subsection we review in more details the properties of the SATW and PSATW models for $d = 1$, which are used in the main text.

1. MSD and ageing of increments

The MSD of both SATW and PSATW models for $d = 1$ are diffusive [10, 11] for $t \to \infty$. We discuss in this subsection the ageing properties of the increments $\langle [x(t+T) - x(T)]^2 \rangle$.

a. SATW

The increments of the SATW have been shown to display scale free ageing[11]. In the limit $t, T \gg 1$ the increments can be written

$$\langle [x(t+T) - x(T)]^2 \rangle = 2D(t/T)\, t, \tag{S5}$$

where the diffusion coefficient $D(t/T)$ has the following finite limits :

$$\begin{cases} D(t/T) \sim D_L(\beta) \text{ for } t \gg T \\ D(t/T) \sim D_s \text{ for } t \ll T. \end{cases} \tag{S6}$$

The long time diffusion coefficient $D_L(\beta)$ is not known analytically ; however, $t \ll T$ it is easy to find that $D_s = 1/2$, because the random walker spends most of its time inside the visited domain $\mathcal{D}_t$, and thus performs a symmetric Pólya walk.

b. PSATW

The increments of the PSATW can be deduced from the above analysis of the SATW[11]. In the long time limit $t \gg T, t_p$ the scaling of the increments is not modified by persistence :

$$\langle [x(t+T) - x(T)]^2 \rangle \sim 2D_L^p(\beta)\, t. \tag{S7}$$

In the regime $t \ll T$ one recovers the classical behavior of persistent random walks. For $t \ll t_p$ the scaling is ballistic

$$\langle [x(t+T) - x(T)]^2 \rangle \sim t^2, \tag{S8}$$

with a cross over to a diffusive regime for $t \gg t_p$

$$\langle [x(t+T) - x(T)]^2 \rangle \sim 2D_s\, t, \tag{S9}$$

with $D_s = e^{2k}/2$.

2. *First-passage properties : persistence.*

We conclude this subsection by reminding the first passage properties of the SATW and PSATW models for $d = 1$. We define the survival probability $S(t)$ as the probability that the walker has not reached a target at time t. The large time behaviour of the survival probability is characterized by a power law decay $S(t) \propto t^{-\theta}$ that defines the persistence exponent[12] θ. The persistence exponent was shown[11] to be given for both the SATW and PSATW by

$$\theta = e^{-\beta}/2. \tag{S10}$$

C. SATW model for $d > 1$

In this subsection, we discuss the properties of the SATW, and more precisely the scaling of the MSD and of its increments; these properties are also expected to apply to the generalised PSATW model in the regime $t, T \gg t_p$. As for $d = 1$, the dynamics of the SATW model is fully defined by the position of the random walker x_t and visited territory $\mathcal{D}_t$ at time t. Qualitatively, the random walker is attracted by $\mathcal{D}_t$: for $d > 1$, the geometry of $\mathcal{D}_t$ is however complex, and only few exact results are available for this process, which has been studied mostly numerically. To determine the scaling of the MSD, a key ingredient is the growth rate of $\mathcal{D}_t$; we provide below scaling arguments to derive these scalings for $d = 3$ and $d = 2$; of note the case $d = 2$ is still debated[13] .

1. *MSD for* $d = 3$

We start with the case $d = 3$. We write

$$\frac{d\langle \mathcal{D}_t \rangle}{dt} \sim \frac{1}{\langle T \rangle_s} \tag{S11}$$

where $\langle T \rangle_s$ is the mean return time to the boundary of $\mathcal{D}_t$ (we identify $\mathcal{D}_t$ and its volume), averaged over all starting positions on the boundary of $\mathcal{D}_t$. Making use of the so called Kac formula for mean return times [14], the scaling of $\langle T \rangle_s$ can be written

$$\frac{1}{\langle T \rangle_s} \sim \frac{\delta\mathcal{D}_t}{\mathcal{D}_t} \tag{S12}$$

where $\delta\mathcal{D}_t$ denotes the boundary of $\mathcal{D}_t$. We now define the walk dimension d_w of the process by the scaling of the MSD: $\langle x^2 \rangle \sim t^{2/d_w}$. We also introduce d_{fc} and α as the fractal dimensions of $\mathcal{D}_t$ and of its boundary $\delta\mathcal{D}_t$ respectively. Using that the length scale of $\mathcal{D}_t$ and $\delta\mathcal{D}_t$ is $\sqrt{\langle x^2 \rangle}$, this yields :

$$\mathcal{D}_t \sim t^{d_{fc}/d_w}, \tag{S13}$$

$$\delta\mathcal{D}_t \sim t^{\alpha/d_w}. \tag{S14}$$

Using the scalings (S13) and (S14) in (S11) and (S12) leads to the following condition of self-consistency:

$$\alpha = 2d_{fc} - d_w. \tag{S15}$$

We first assume that the strength of the self interaction is small enough ($|\beta|$ small) so that $\mathcal{D}_t$ has the same scaling properties as for a classical symmetric random walk for $d = 3$. It is known that the territory explored by a classical random walk is characterized by $\alpha = d_{fc} = 2$. Equation (S15) is then satisfied for $d_w = 2$. This scaling argument suggests that for $|\beta|$ small, the SATW displays a normal diffusive scaling, which is indeed observed numerically. Let us now assume that for $|\beta|$ large the dimension of $\mathcal{D}_t$ is modified ; more precisely we assume that $\mathcal{D}_t$ is a smooth volume of dimensions $d_{fc} = d = 3$ and $\alpha = d_{fc} - 1 = 2$. Equation (S15) then leads to $d_w = 4$. This scaling argument therefore suggests the existence of a sub-diffusive regime with $\langle x^2(t) \sim\rangle \sim t^{1/2}$. This subdiffusive regime is indeed observed numerically. Of note, the transition between the diffusive and subdiffusive regimes has not yet been characterized[13].

2. *MSD for* $d = 2$

In principle, the above scaling analysis can be reproduced for $d = 2$. For $|\beta|$ large, it suggests a subdiffusive regime $\langle x^2(t) \rangle \sim t^{2/3}$, which is indeed observed numerically. The behaviour for $|\beta| \to 0$, and in particular the transition to a diffusive regime is however still debated [13].

3. Increments

We discuss in this subsection the aging properties of the increments $\langle [x(t+T) - x(T)]^2 \rangle$.

a. subdiffusive regime ($|\beta|$ large)

The numerical analysis of the process suggests the following behaviours in the sub-diffusive regime for both $d = 2$ and $d = 3$ ($|\beta|$ large). For $t \gg T$ the scaling of the MSD is obtained : $\langle [x(t+T) - x(T)]^2 \rangle \sim t^{2/d_w}$, where we recall that $d_w = 4$ for $d = 3$ and $d_w = 3$ for $d = 2$. For $t \ll T$, the random walker is within $\mathcal{D}_t$, which is a smooth d–dimensional volume ; the process is thus a classical diffusion and $\langle [x(t+T) - x(T)]^2 \rangle \sim 2dD_s t$.

b. diffusive regime ($|\beta|$ small and d=3)

Our numerical analysis suggests that the increments are asymptotically stationary for $d = 3$ in the diffusive regime:

$$\langle x^2(t) \rangle \sim 2D_L t. \tag{S16}$$

4. Impact on exploration : type problem and FPT properties

The existence of a sub diffusive regime for $d = 2, 3$ has drastic consequences on space exploration. Indeed, in the subdiffusive regime one hase $d_w > d$ and exploration is called compact for $d = 2, 3$, as opposed to the classical symmetric Polya walk, which is non compact for $d = 3$ and only marginally compact for $d = 2$. For compact random walks, each site of the lattice is visited ultimately infinitely many times, and all sites are eventually visited with probability 1. Qualitatively, the random walker explores densely and exhaustively its neighborhood, while $\mathcal{D}_t$ grows smoothly with "small" fluctuations. In particular the resulting survival probability is observed to decay faster than any power law, so that the persistence exponent is effectively infinite in this regime.

5. Comparison to experimental data : continuous time PSAW model

To compare quantitatively the PSAW model to experimental cell trajectories, it is needed to effectively take into account both the fluctuations of cell velocities and cell/cell variability. To take into account fluctuations of cell velocities, we define a continuous time version of the PSAW model as follows. The waiting times between successive hopping events are assumed to be independent and identically distributed according to an exponential distribution of average value $\langle T \rangle = 0.6h$. This choice of exponential distribution and the value $\langle T \rangle$ are deduced from experimental data. To take into account cell/cell variability, for each trajectory generated numerically the parameter k (and thus $\beta = -2(k+1)$) is drawn from a normal distribution of standard deviation ≈ 0.5 which reflects the variability observed in experiments. The effect of both fluctuations of cell velocities and cell/cell variability is shown in figure 20. In the discrete time model and in absence of variability of k, a local maximum of the increments is observed ; it corresponds to reversal events at the edge of the visited span that occur at a time that displays small fluctuations. Taking into account fluctuations of cell velocities and cell/cell variability, fluctuations of such reversal times increase and the maximum of the increments disappears.

III. Supplementary movies

Supplementary movie 1 Oscillating MDCK cell on a 20 μm track. Phase contrast images. Scale bar 100 μm.

Supplementary movie 2 Static MDCK cell on a 20 μm track. Phase contrast images. Scale bar 100 μm.

Supplementary movie 3 Oscillating MDCK cell on a 20 μm track. Fluorescence intensity of PBD-YFP. Scale bar 50 μm.

Supplementary movie 4 MDCK cell on a 20 μm track, alternating between oscillatory and static phases. Fluorescence intensity of PBD-YFP. Scale bar 50 μm.

Supplementary movie 5 MDCK cell on a control (top, same movie as Supplementary movie 1) and a conditionned (bottom) 20 μm track. Scale bars 100 μm.

IV. Supplementary Figures

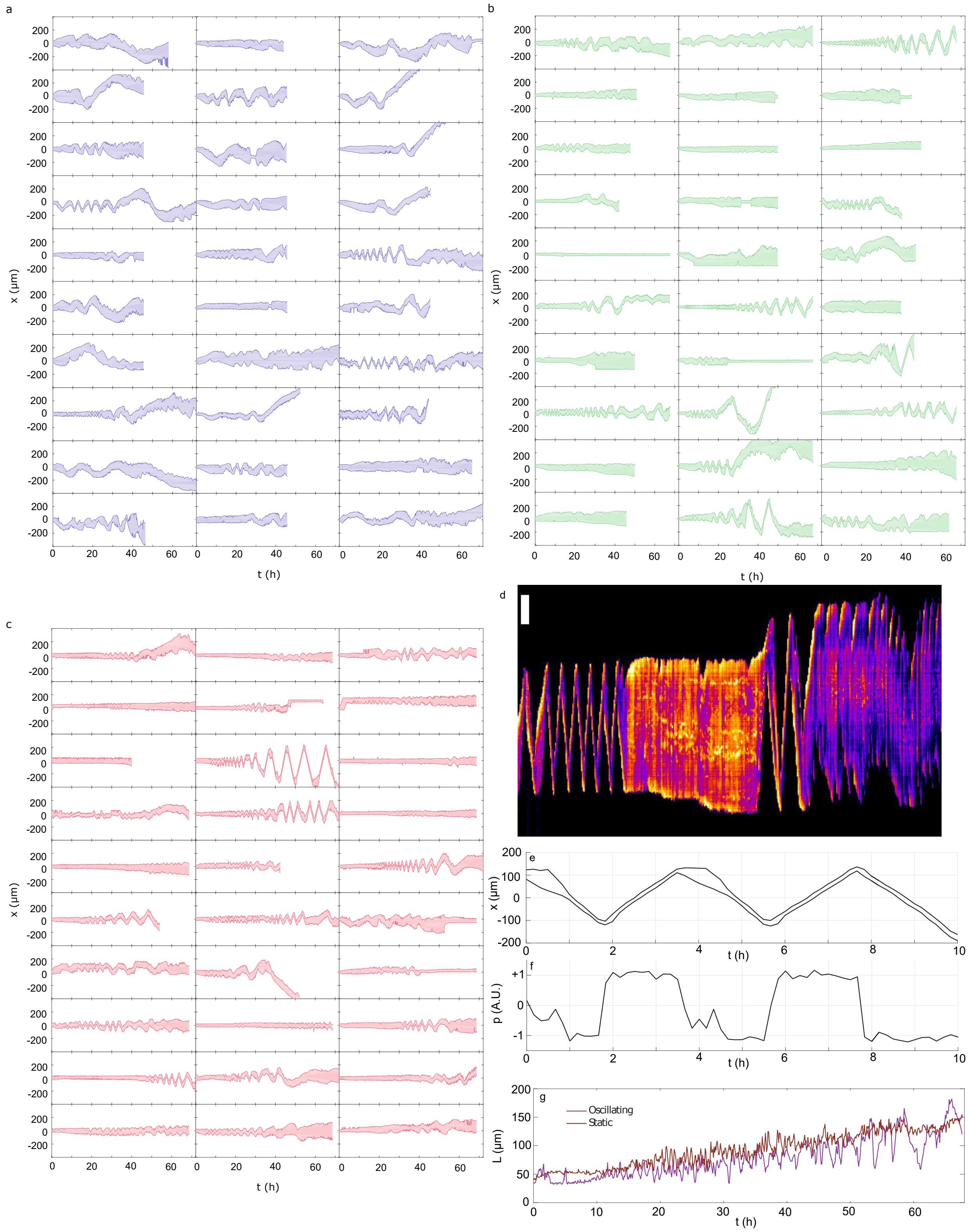

Supplementary Figure 1. **a–c.** 30 randomly sampled trajectories of MDCK cells on linear tracks of width $W = 10$ (**a**), 20 (**b**) and 50 μm (**c**) respectively. The vertical x scale is 200 μm per tick, the horizontal t scale is 10 h per tick (full ranges, $t \in [0; 72]$ h, $x \in [-400; 400]$ μm). **d.** Cell length as a function of time for the two examples showed in Figure 1d-e. **e.** Edge position as function of time for the PDB-YFP MDCK cell showed in Figure 1g. **f.** Polarity p as a function of time for the PBD-YFP MDCK cell showed in Figure 1g. p is computed as the moment of the spatial distribution of PBD signal intensity in the cell, with respect to the cell centre-of-mass. **g.** Kymograph of the PBD signal in a MDCK cell undergoing oscillations and static spreading in alternation. Scale bar 20 μm

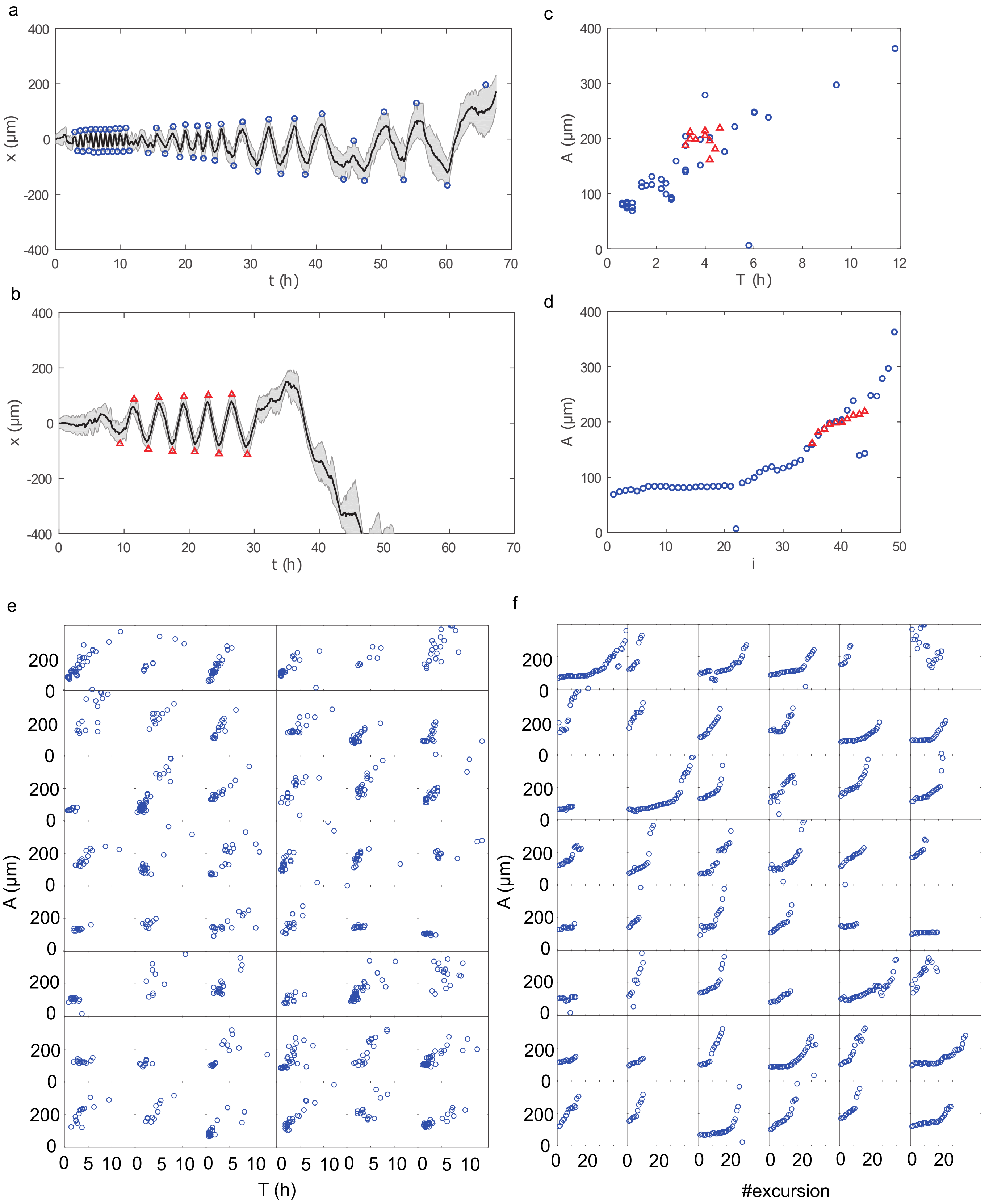

Supplementary Figure 2. Oscillation analysis. **a-b.** Two cell kymographs with the detected peaks marked as blue circles (**a**) and red triangles (**b**). **c.** Amplitude versus period of detected oscillations for the two examples in a (blue circles) and b (red triangles). d. Sequence of amplitude of oscillations in the two examples in a and b. The data from panel b have been shifted to the right to show that their rate of increase is similar to that of oscillations of same amplitude in a. **e.** Amplitude of oscillations as a function of their time period. Each plot corresponds to an individual trajectory. $W = 20\,\mu\mathrm{m}$. **f.** Time series of the amplitude of oscillations. Same data as in **e**.

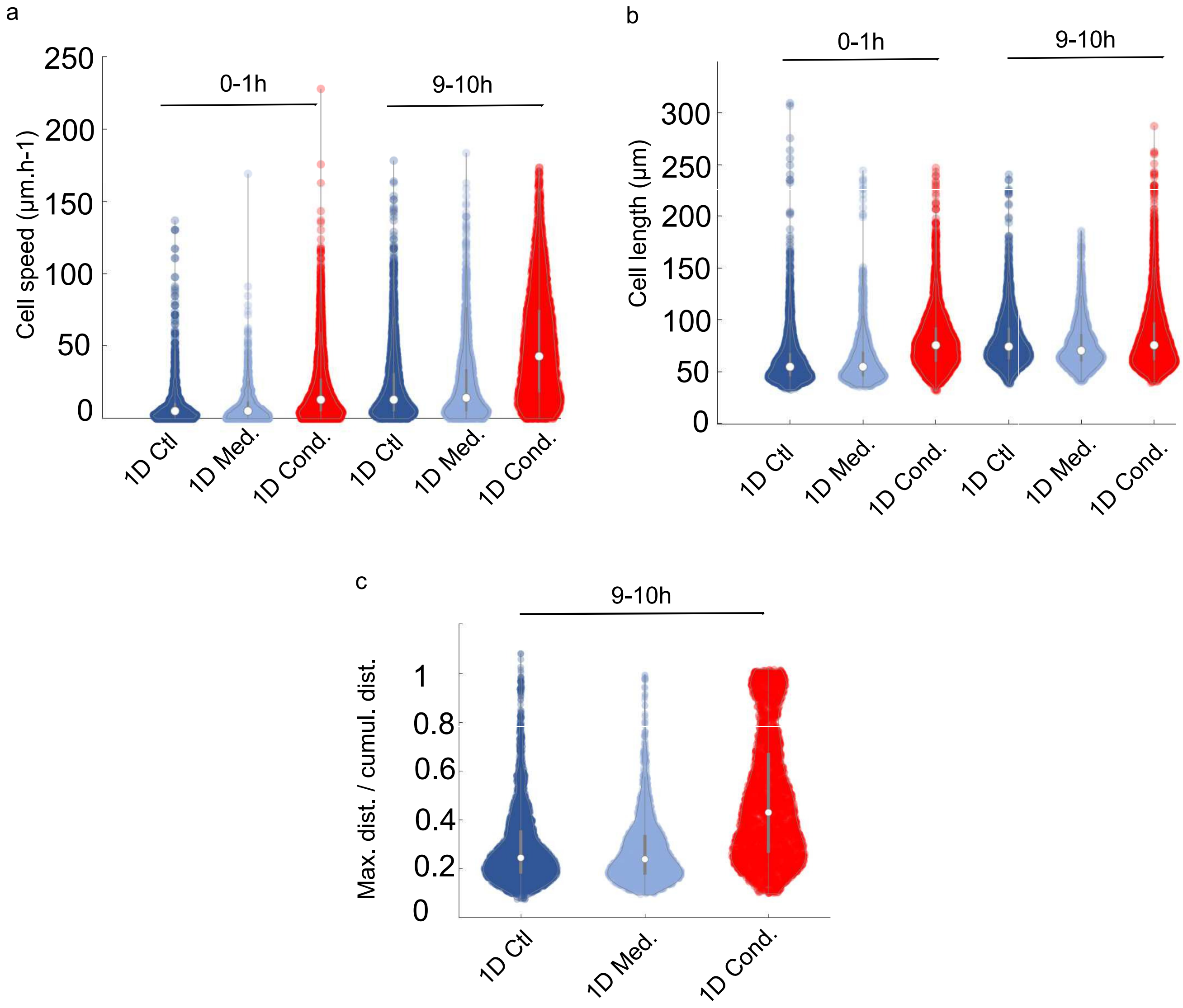

Supplementary Figure 3. Effect of conditioning on cell characteristics. **a.** Instantaneous cell speed for MDCK cells on control, control with medium and conditioned linear substrates, at early $(0-1\,\mathrm{h})$ and later $(9-10\,\mathrm{h})$ times. **b.** Cell length for MDCK cells on control, control with medium and conditioned linear substrates, at early $(0-1\,\mathrm{h})$ and later $(9-10\,\mathrm{h})$ times. **c.** Persistence of the movement, defined as the ration of maximum distance to origin to cumulative path length, at $t = 9 - 19\,\mathrm{h}$, for MDCK cells on control, control with medium and conditioned substrates. $n = 355$, 238, 429 trajectories from 3 independent experiments.

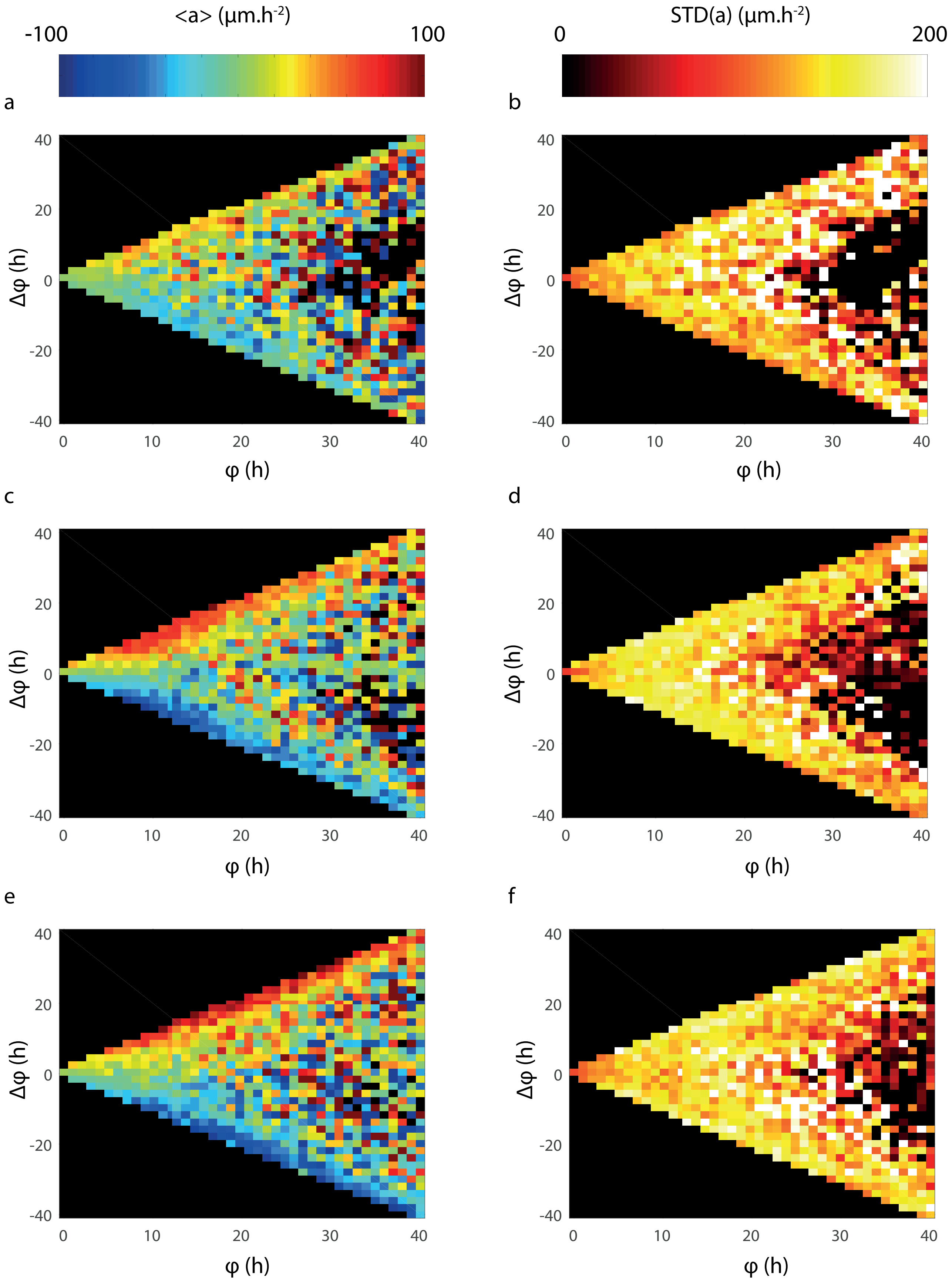

Supplementary Figure 4. $\varphi - \Delta\varphi$ phase space. Average (**a, c, e**) and standard deviation (**b, d, f**) of the acceleration as a function of φ and $\Delta\varphi$ for cells on tracks of width 10 (**a,b**), 20 (**c,d**) and 50 (**e,f**) μm.

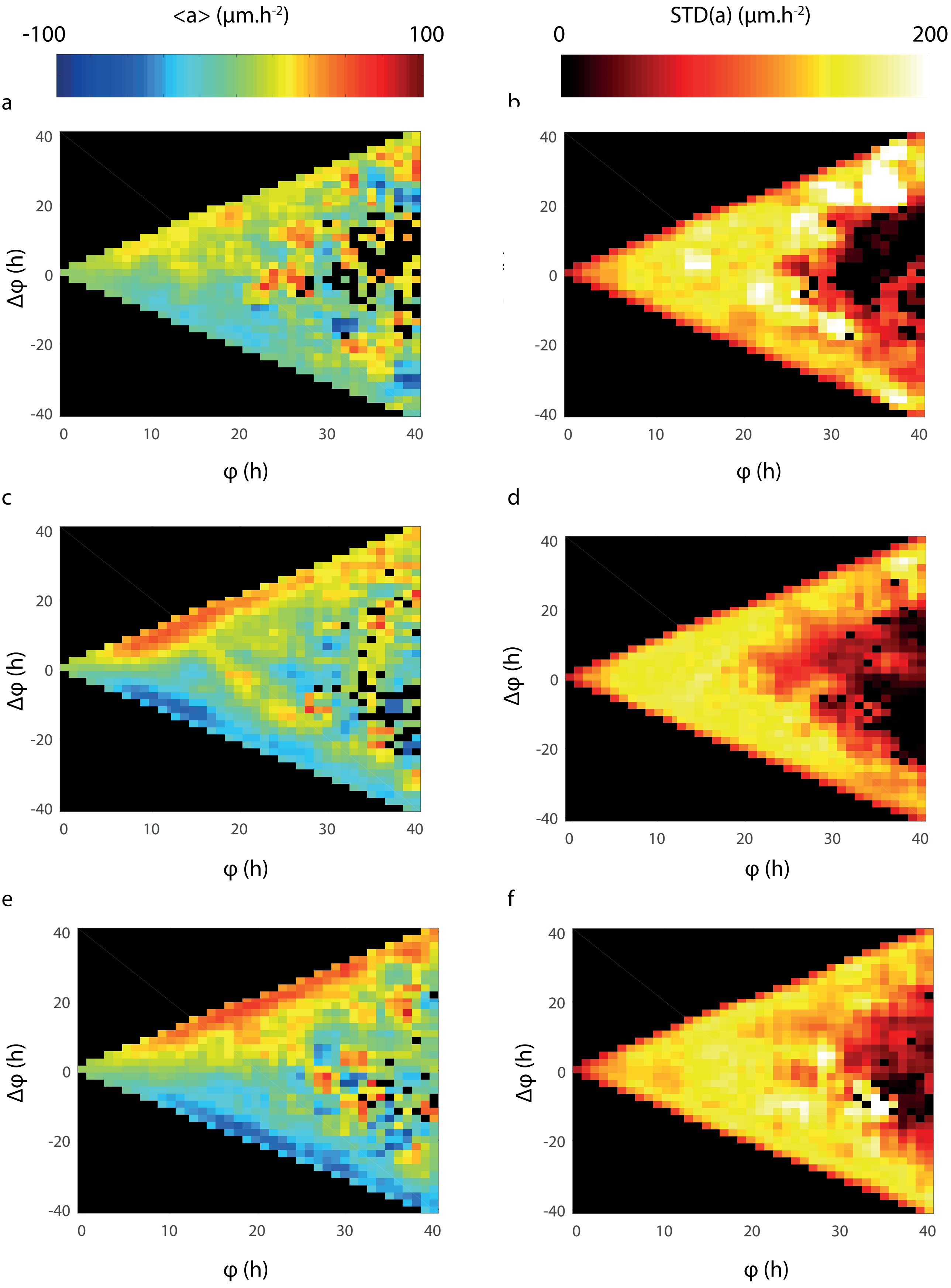

Supplementary Figure 5. $\varphi - \Delta\varphi$ phase space. Average (**a, c, e**) and standard deviation (**b, d, f**) of the acceleration as a function of φ and $\Delta\varphi$ for cells on tracks of width 10 (**a,b**), 20 (**c,d**) and 50 (**e,f**) μm. Same data as in Supplementary Figure 4, smoothed over 3x3 squares.

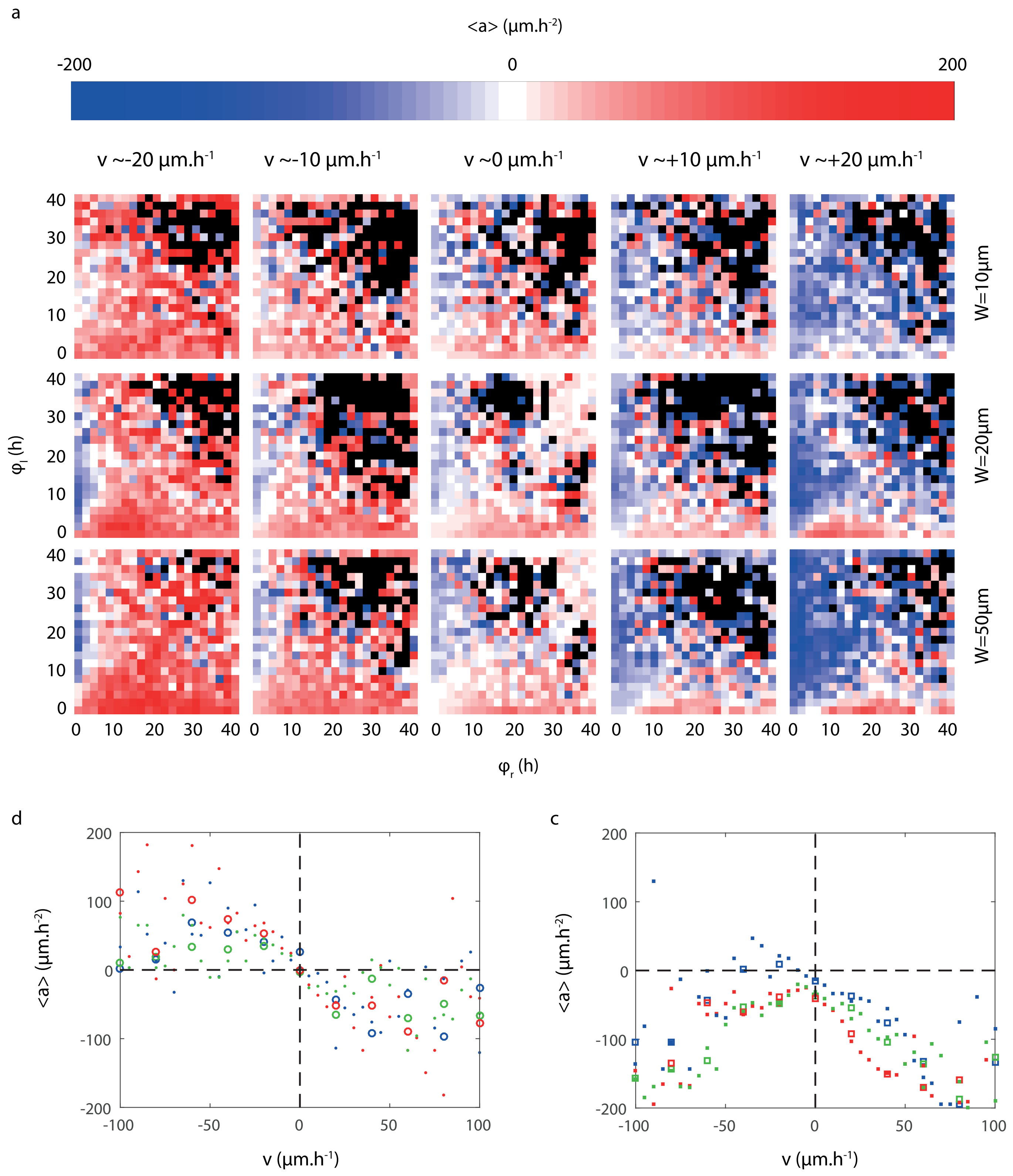

Supplementary Figure 6. $\varphi_l - \varphi_r$ phase space. **a.** Average acceleration as a function of v, φ_l and φ_r for cells on tracks of various widths. **b.** $\langle a \rangle$ as a function of v measured near the $\varphi_l = \varphi_r$ diagonal. **c.** $\langle a \rangle$ as a function of v measured near the $\varphi_r = 0$ axis (data near the $\varphi_l = 0$ axis also pooled after symmetrisation).

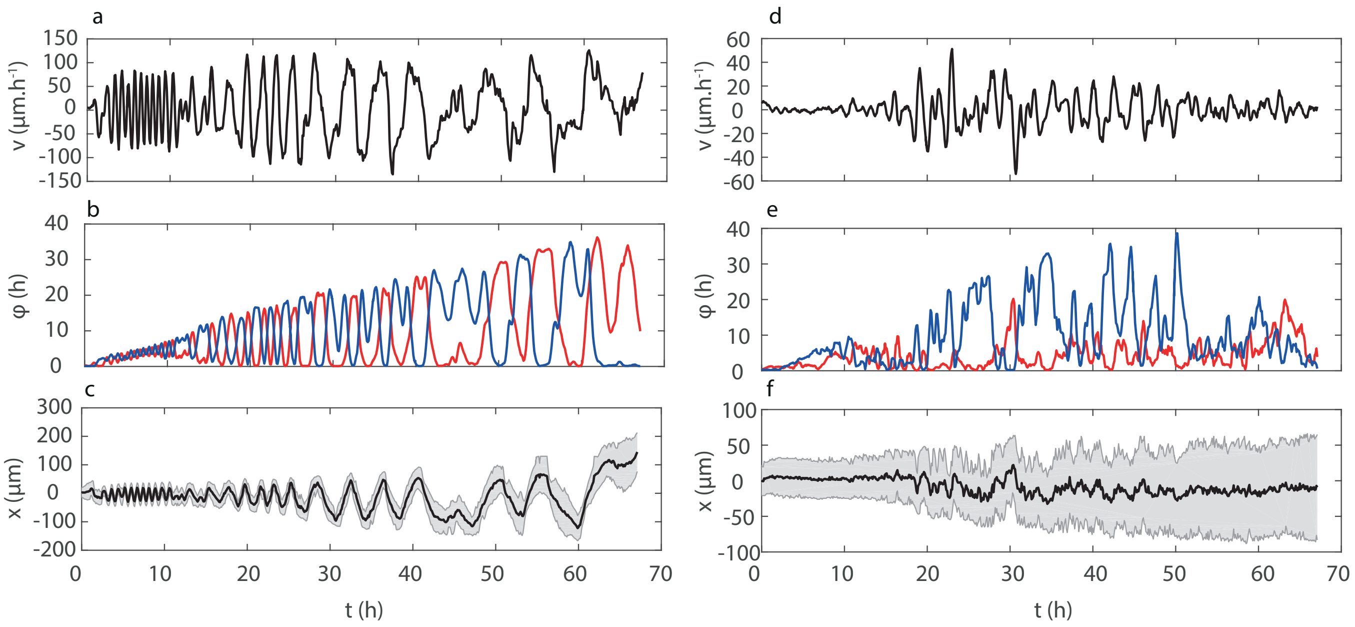

Supplementary Figure 7. Motion in the $(v, \varphi_l, \varphi_r)$ phase space. Velocity (**a,d**), footprint values at the cell ends (**b,e**) and kymographs (**c,f**) for the two cells shown in Figure 1d-e of the main text.

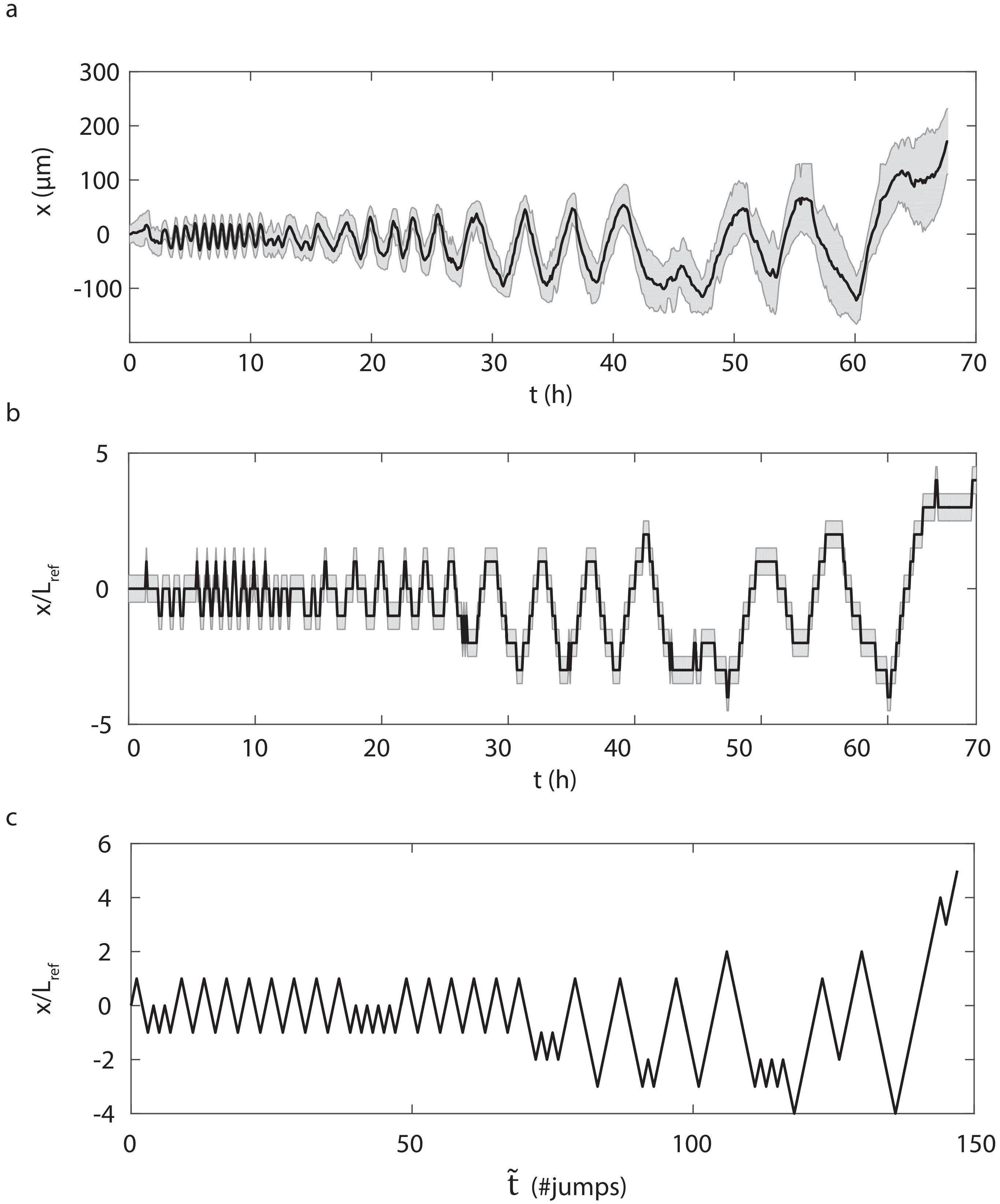

Supplementary Figure 8. Discretisation procedure. **a.** Original cell kymograph. **b.** Cell kymograph after discretisation of space using the cell's minimal length as K_{ref}. **c.** Cell kymograph after keeping only jump events as time steps.

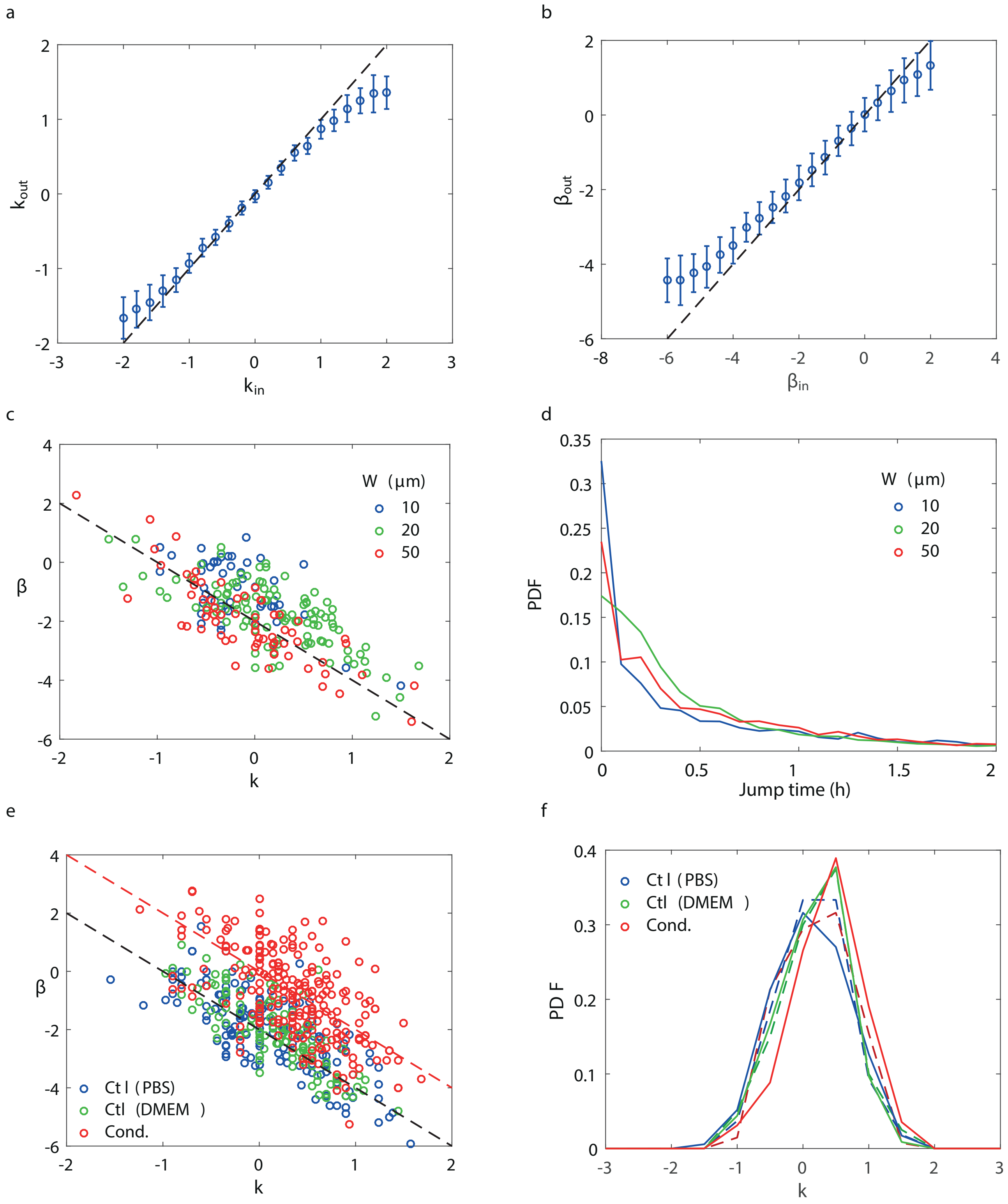

Supplementary Figure 9. PSAW analysis. **a.** Measured k values as a function of input k in simulated trajectories analysed with the discretisation procedure. **b.** Measure β values as a function of input β values in simulated trajectories analysed with the discretisation procedure. **c.** Experimental β versus k from cells on tracks of different widths. $\beta = -2(k+1)$ fit (dashed line). **d.** Distribution of jump times measured during the discretisation procedure for cells on tracks of different widths. **e.** Expermental β versus k for cells on control or conditioned substrates on lines of 20 μm in width. $\beta = -2(k+1)$ (black) and $\beta = -2k$ (red) fits (dashed lines). **f.** Distribution of measured k for cells on control or conditioned substrates, with two definitions of L_{ref}: minimal (solid lines) or median (dashed lines) of the cell length. In panels **a**-**b**, mean and standard deviation obtained from 1000 simulated trajectories per parameter set are shown.

a

b

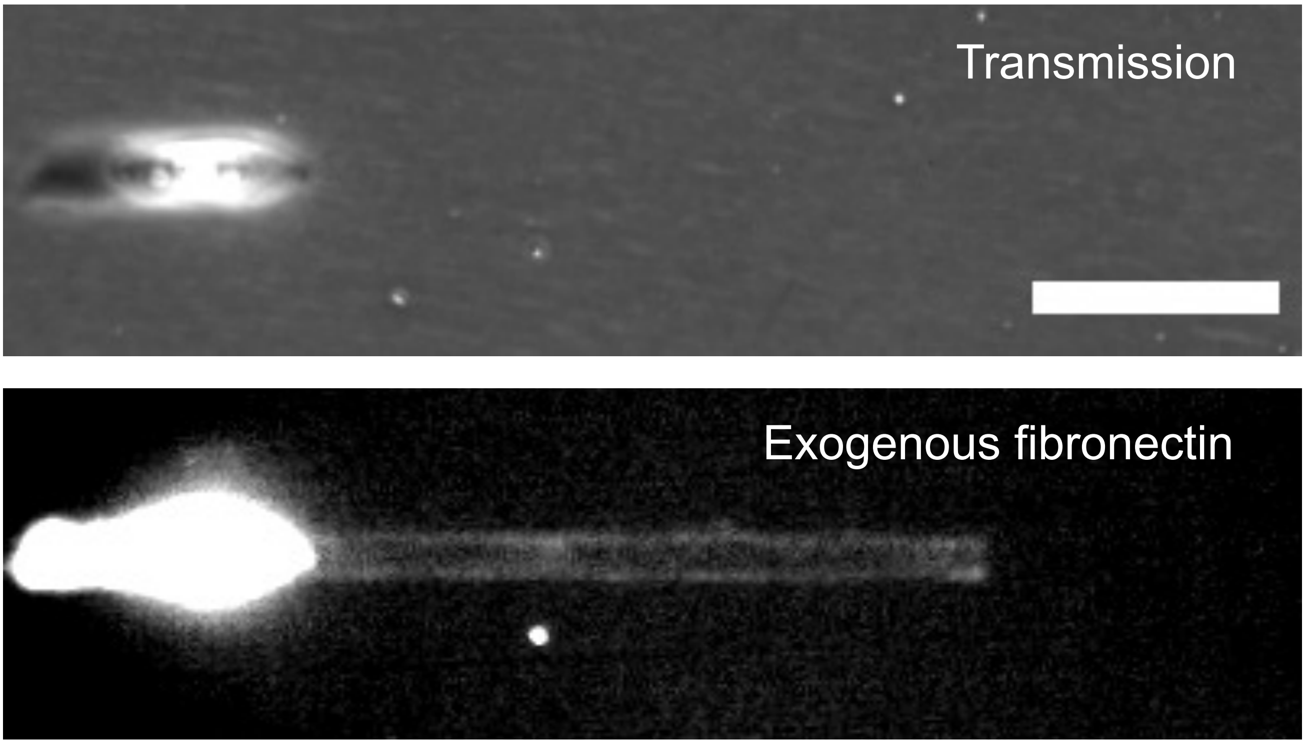

Supplementary Figure 10. Exogeneous fibronectin is captured and deposited by moving cells. **a.** Phase contrast image of an isolated cell on a $20\ \mu\mathrm{m}$ line after $48\ \mathrm{h}$ incubation with labeled fibronectin and fixation. **b.** Corresponding fluorescence image of the labeled fibronectin, showing signal along the line close to, but away from the cell. Scale bar $100\ \mu\mathrm{m}$. Deposited exogenous fibronectin was observed in 2 independent experiments, although with different observation set-ups.

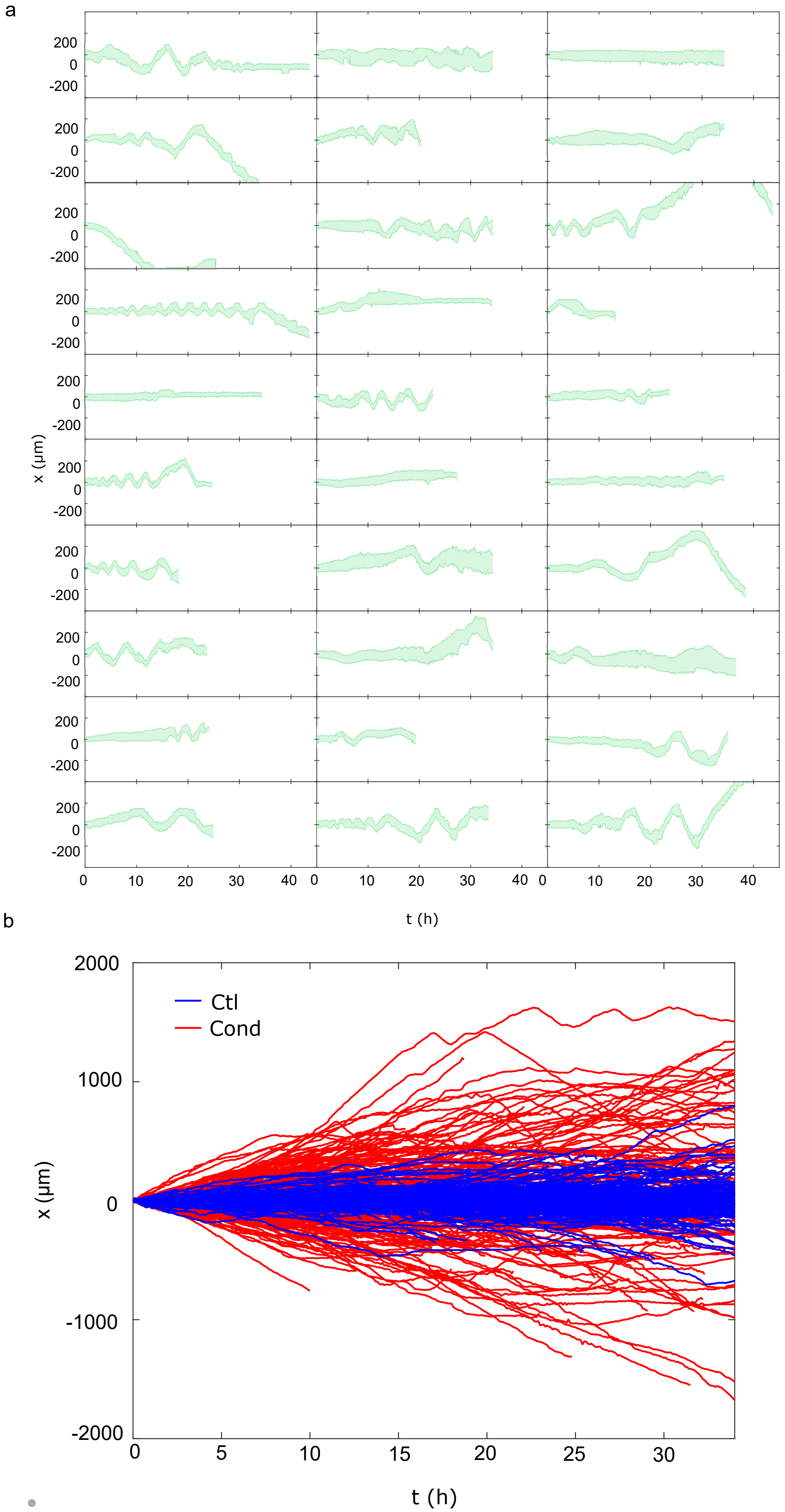

Supplementary Figure 11. Caco2 cells also exhibit footprint-related oscillations. **a.** Sample kymographs of isolated Caco2 cells on 20 µm line patterns. **b.** Trajectories of Caco2 cells on control (Ctl, blue) and conditioned (Cond., red) 1D substrates. Resp. $n = 246$ and 216 trajectories for Ctl and Cond. substrates from 3 independent experiments.

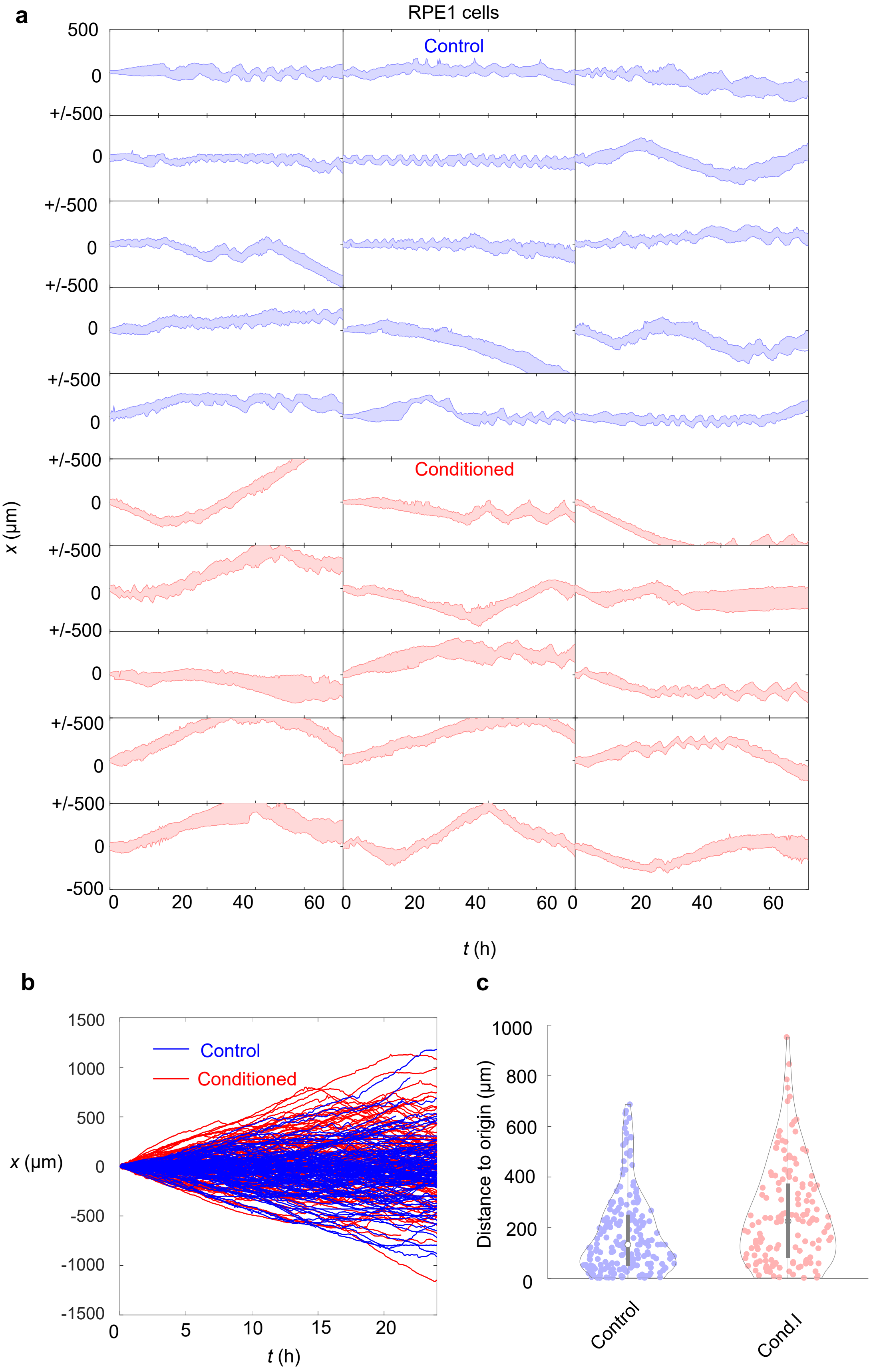

Supplementary Figure 12. RPE1 cells exhibit oscillations, although less consistently than MDCK cells. **a.** Sample kymographs of RPE1 cells on control (blue, 15 topmost graphs), and conditioned (red, 15 lower graphs) lines. These graphs are not statistically significant but they were chosen to show that some cells exhibit an oscillatory behaviour. **b.** All trajectories of RPE1 cells on control (blue) and conditioned (red) lines. There is a noticeable but slight difference in displacements between both conditions. **c.** Distance of cells to the origin after 16 h. $p = 1.10^{-3}$. Resp. $n = 198$ and 146 trajectories for Ctl and Cond. substrates from 2 independent experiments.

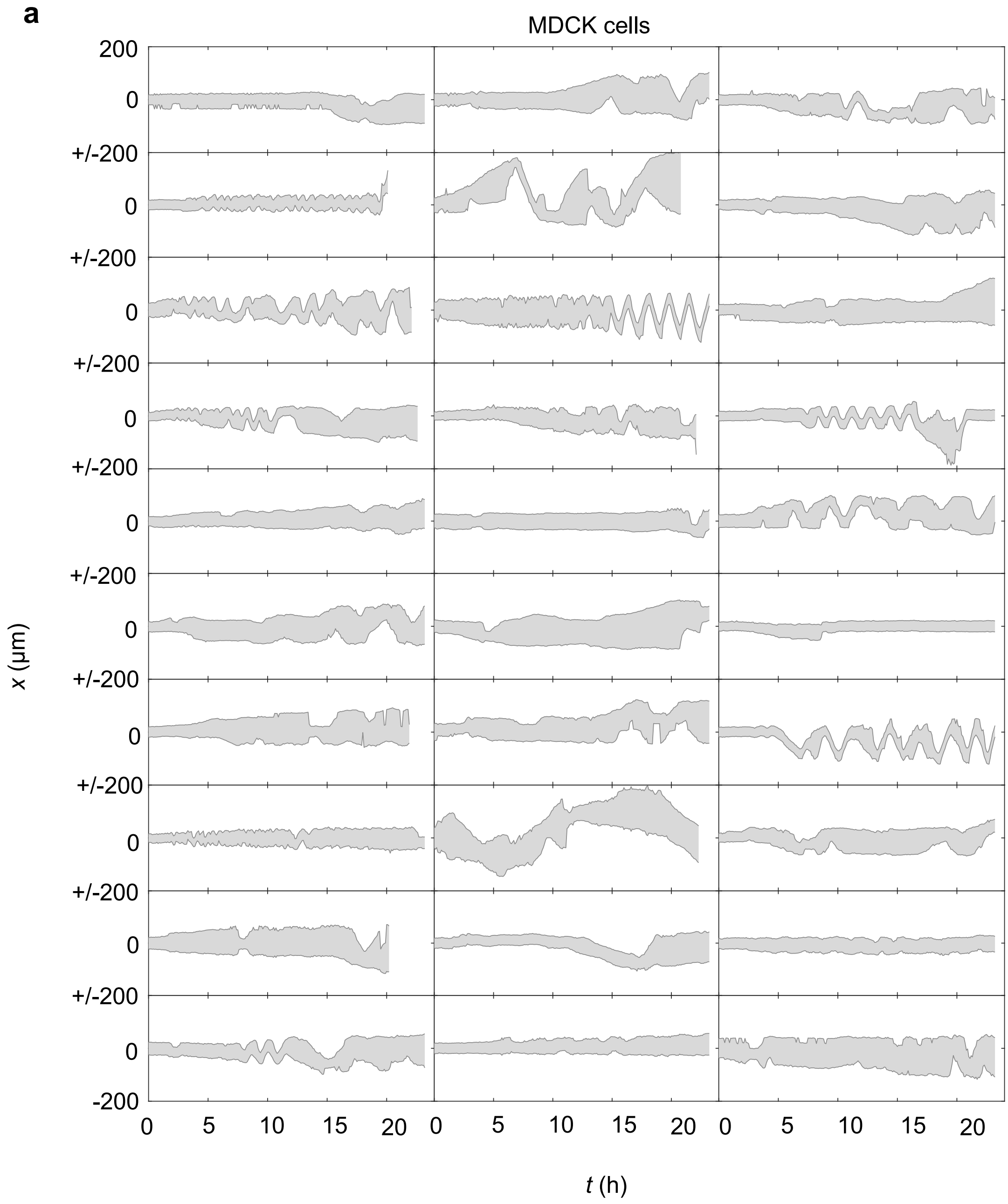

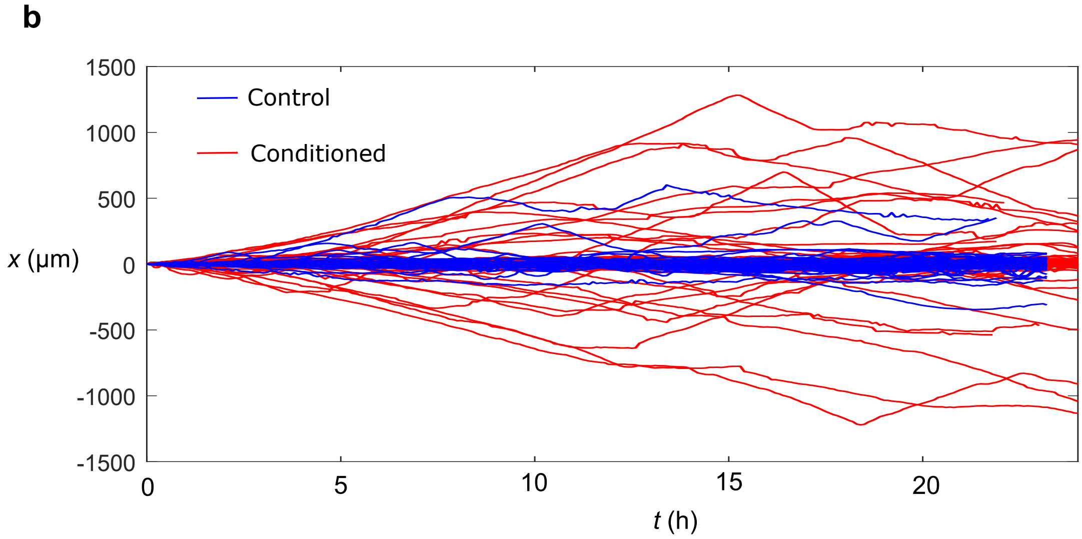

Supplementary Figure 13. Mitomycin C treatment does not affect cell migration. **a.** Randomly sampled kymographs of MDCK cells plated on 20 μm lines, without prior treatment with mitomycin C. **b.** All cell tracks for untreated MDCK cells on control (blue) and conditioned (red) substrates. Resp. $n = 94$ and 74 trajectories for Ctl and Cond. substrates from 2 independent experiments.

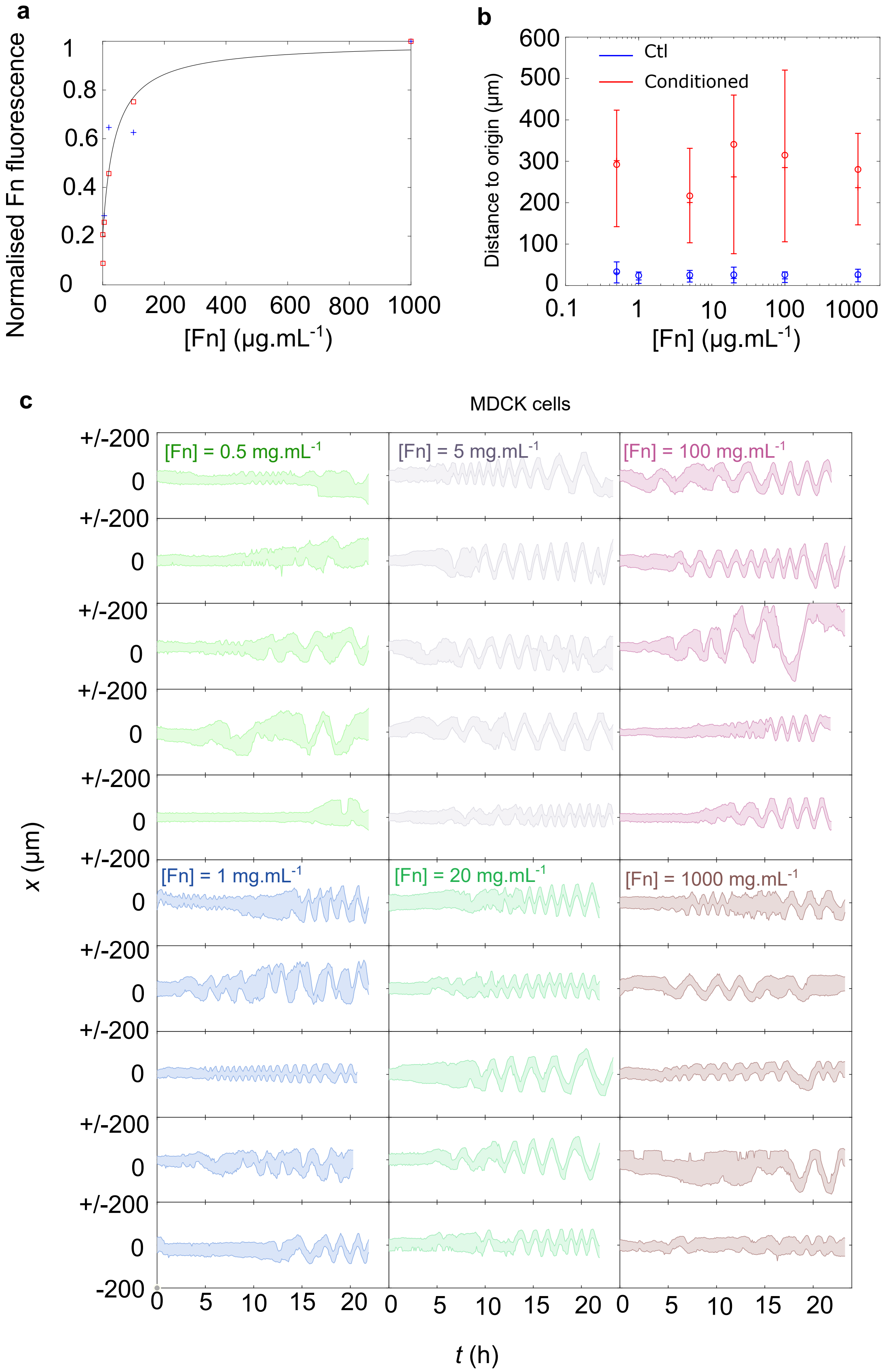

Supplementary Figure 14. Patterning concentration of fibronectin is not a determinant of the oscillations. **a.** Normalised fluorescence intensity of line patterns prepared with various concentrations of Cy3-labelled fibronectin. Plus (+) and square signs refer to two repeats. The solid line is a fit with an adsorption isotherm. **b.** Distance to origin after 16 h for cells plated on control (blue) and conditioned (red) lines prepared with various concentrations of fibronectin. (+) median, (o) mean, and lower and upper quartiles are shown. **c.** Sample kymographs of cells plated on (control) lines prepared with various concentrations of fibronectin display the same oscillatory behaviour. $n = 7, 19, 57, 60, 53, 18$ trajectories for control and $n = 6, 0, 24, 33, 32, 41$ for conditionned subtrates respectively, in order of ascending Fn concentration.

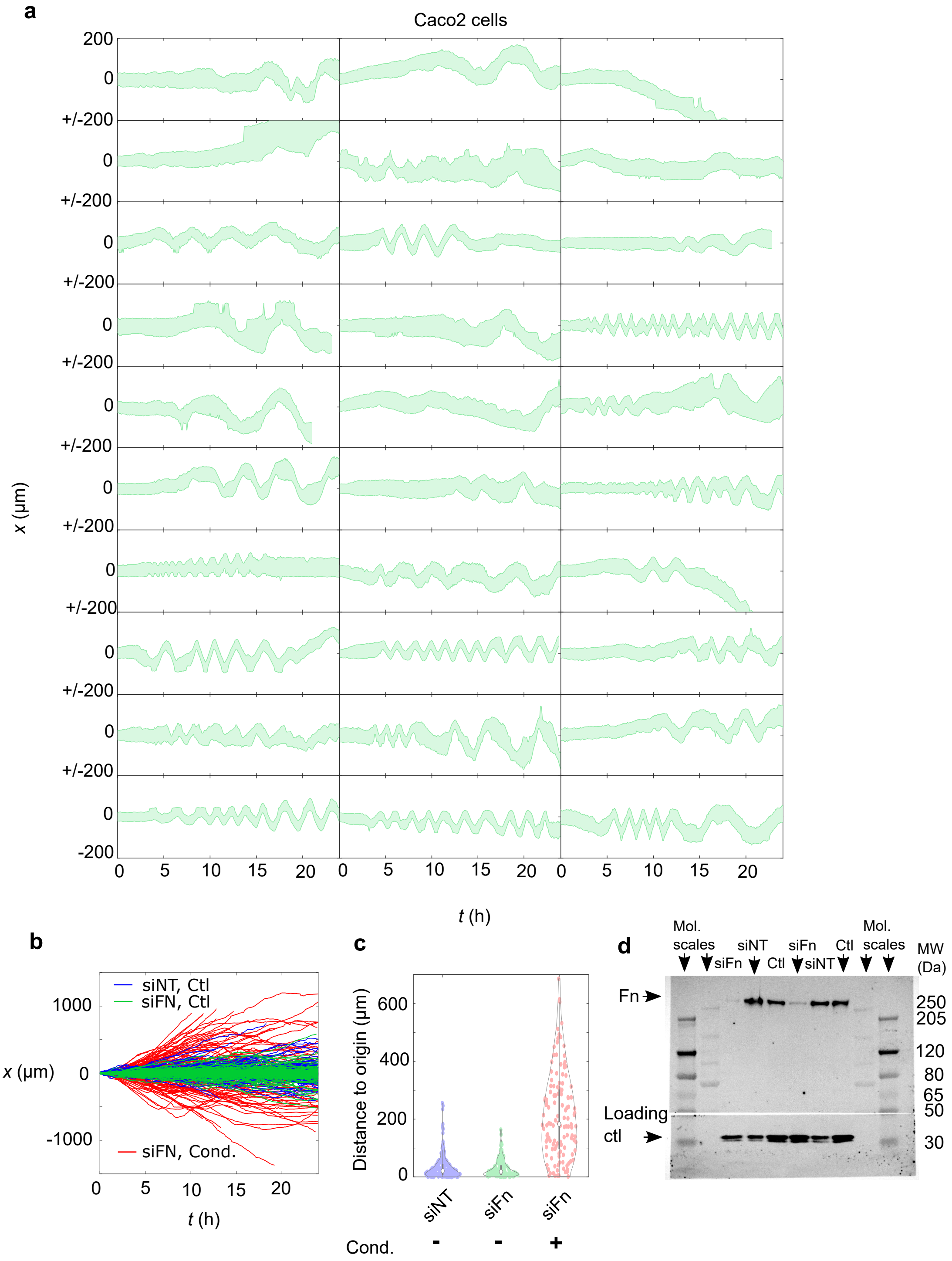

Supplementary Figure 15. Knock-out of fibronectin production alone is not enough to suppress oscillatory behaviour. **a.** Sample trajectories of Caco2 cells treated with siRNA targeted against fibronectin. **b.** All trajectories of Caco2 cells treated with non-targetting (blue) or anti-Fn (green and red) siRNA, plated on control (blue and green) or conditioned (red) substrates. **c.** Distance to origin after 8 h for Caco2 cells in the same conditions as in **b** (see figure legend). **d.** Raw Western blot showing a 95% depletion of fibronectin protein in cells treated with siRNA targeting Fn (siFN). siNT: cells transfected with non-targetting siRNA; Ctl.: untransfected cells. Mol. scales: molecular weight markers. Loading control GAP-DH. The protein content could be assessed only in one repeat of the experiment due to the low quantity of reagents available but the transfection procedure was conducted exactly similarly in 2 independent experiments. $n = 210, 134$ and 130 trajectories for (siNT / control substrate), (siFn / control substrate) and (siFn / conditionned substrate) respectively from 2 independent experiments.

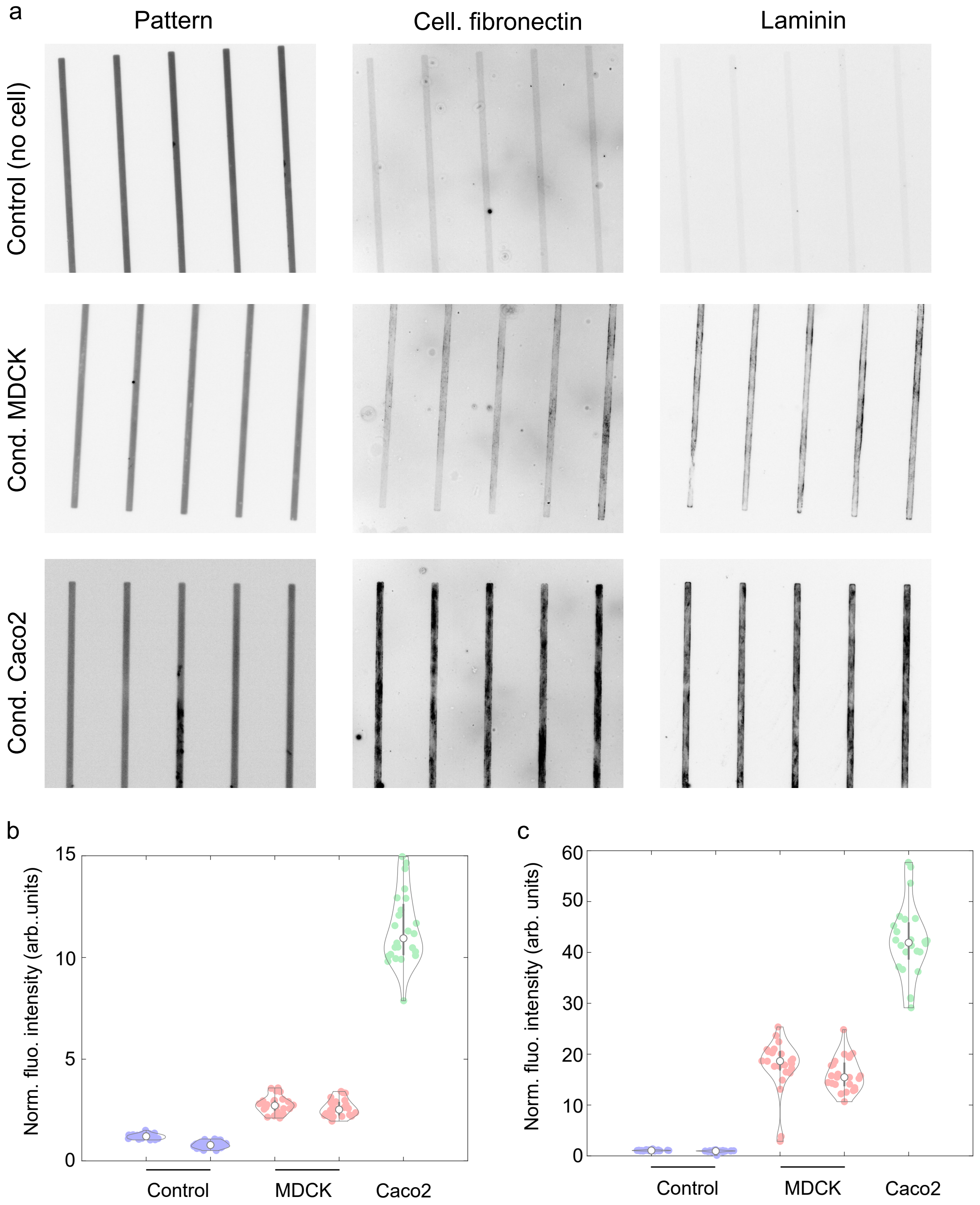

Supplementary Figure 16. Both MDCK and Caco2 cells deposit extra-cellular matrix components on their way. **a.** Fluorescence images of fixed control and (both MDCK- and Caco2-) conditioned substrates. Pattern is seen using Cy5-labelled (plasma) fibronectin, while cellular fibronectin and laminin are visualised by immuno-staining. **b-c.** Quantification of the fluorescence intensity in both fibronectin (**b**) and laminin (**c**) channels, normalised by the mean fluorescence on control lines. Note that the difference between MDCK and Caco2 may not reflect a quantitative difference in protein surface density, the used antibodies were directed against human proteins, and their affinity with their dog orthologs might not be equivalent. $n = 24$ pictures (60 line patterns in total) for each independent experiment.

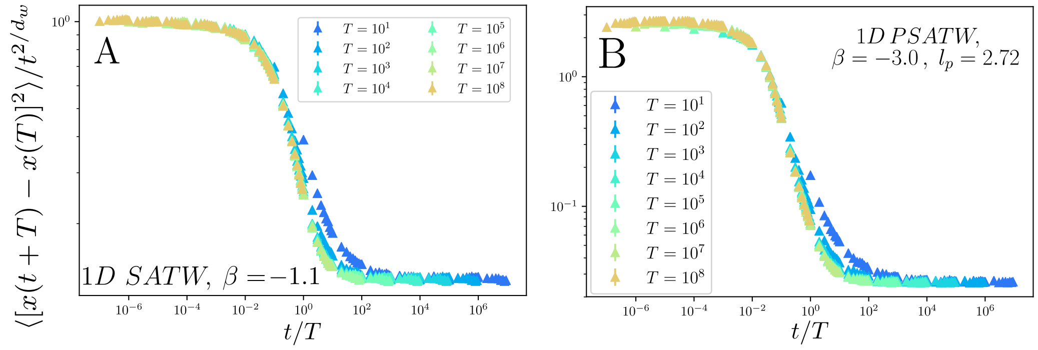

Supplementary Figure 17. Aging of the increments for the SATW (**a**) and the PSATW (**b**), normalized by the expected diffusive scaling at long times. Each curve corresponds to a fixed value of T.

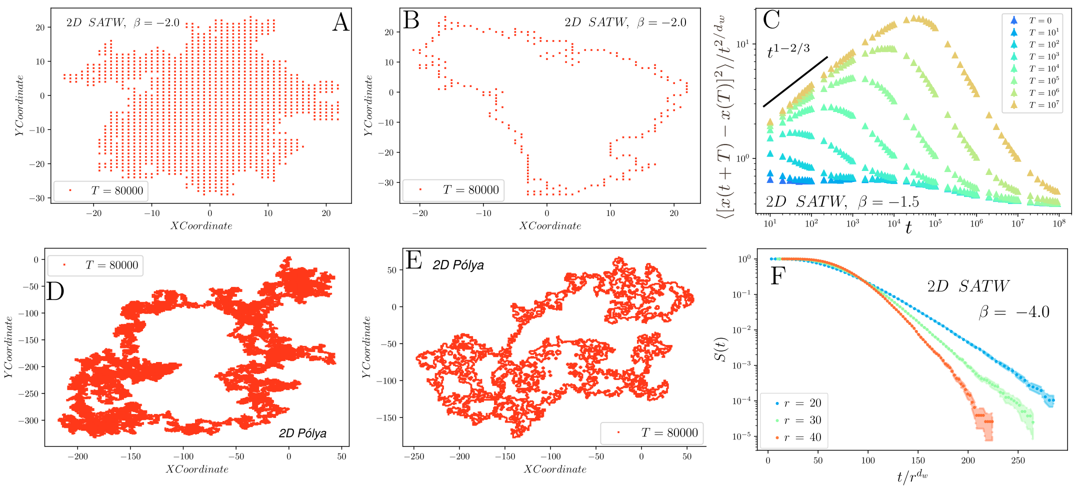

;

Supplementary Figure 18. Area visited and aging for the SATW in dimension 2, and comparison with a simple random walk. **a, d.** Area visited in dimension 2 for a subdiffusive SATW (**a**) and for a simple random walk (**d**). The set of visited sites $\mathcal{D}_t$ for the SATW grows as a 2–dimensional smooth compact set with an area that scales as t^{4/d_w} contrary to the simple random walk where $\mathcal{D}_t$ as a null area in the continuum limit. **b, e.** Boundary of $\mathcal{D}_t$ for the SATW random walk (**b**) and for a simple random walk (**e**). The dimensions of the boundary sets are $\alpha_p = 1$ for the SATW and $\alpha_p = 2$ for a simple random walk. **c.** Aging of the increments for the subdiffusive SATW normalized by the expected diffusive scaling at long times. Each curve corresponds to a fixed value of T. Note that the increments are diffusive for $t \ll T$, when the walker is mostly located inside $\mathcal{D}_t$ and performs a simple symmetric nearest random walk. **f.** Survival probability has a function of time for different values of r. For a sufficiently large $|\beta|$, the survival probability decays faster than exponentially (we observe a decay consistent with an exponential form), so that $\theta = \infty$.

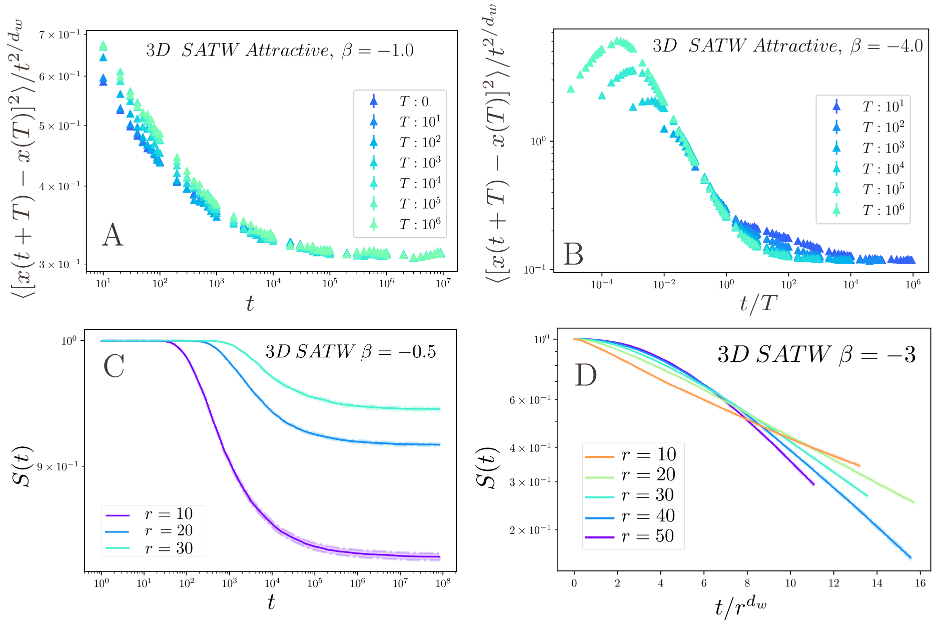

Supplementary Figure 19. First passage properties and aging of the SATW in dimension 3. **a-b.** Aging of the increments for the SATW (normalized by the expected diffusive scaling at long times). Each curve corresponds to a fixed value of T. The increments are stationary at long times at low $|\beta|$ for the diffusive regime (**a**) contrary to the subdiffusive case (**b**) where a diffusive regime is recovered for $t \ll T$. **c-d.** Survival probability has a function of time (log scale) for different values of r. In the diffusive case (**c**), the survival probability tends to a non-zero constant which depends on r : exploration is thus non compact. Conversely for a sufficiently large $|\beta|$, in the subdiffusive case (**d**), the process performs a compact exploration with an exponential-like decay of the survival probability.

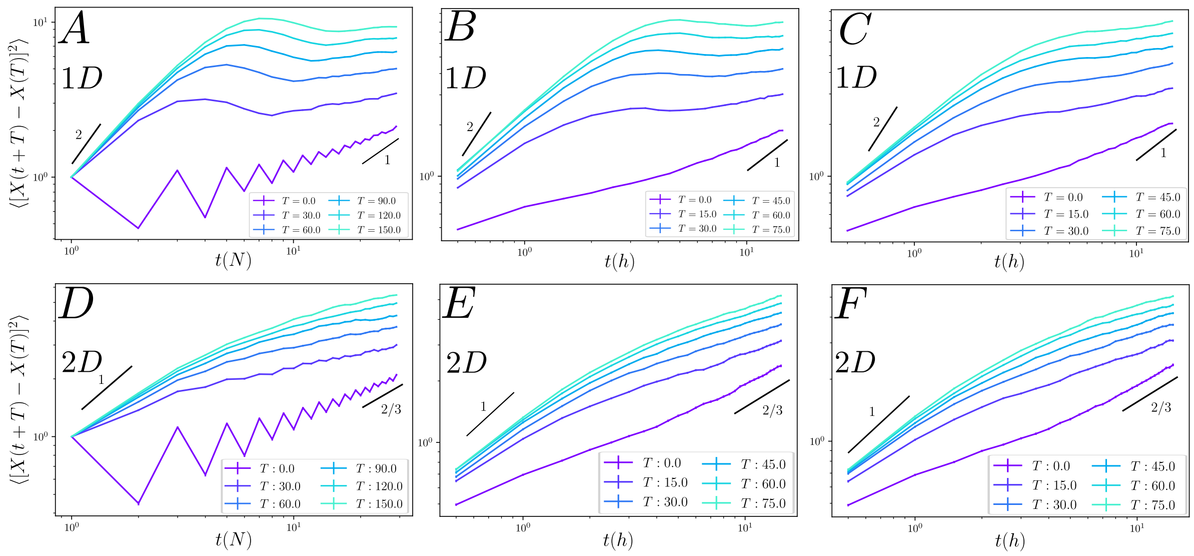

Supplementary Figure 20. Increments of the discrete time PSAW, continuous time PSAW, and continuous time PSAW with random parameters k, β. Each curve corresponds to a fixed value of T in dimension 1 (**a, c**) and 2 (**d, f**). **a, d**: discrete time PSAW (t is given in number of steps) with fixed parameters k, β. **b, e**: continuous time PSAW with fixed parameters k, β. Waiting times are drawn form an exponential distribution of mean $\langle T \rangle = 0.6h$. **c, d**: the parameter k (and thus $\beta = -2(k+1)$) is drawn from a normal distribution with $\langle k \rangle = 0.5$ and standard deviation 0.5.

[1] David B. Brückner, Alexandra Fink, Christoph Schreiber, Peter J. F. Röttgermann, Joachim O. Rädler, and Chase P. Broedersz. Stochastic nonlinear dynamics of confined cell migration in two-state systems. *Nature Physics*, 15(6):595–601, June 2019. Number: 6 Publisher: Nature Publishing Group.

[2] Burgess Davis. Reinforced random walk. *Probability Theory and Related Fields*, 84(2):203–229, June 1990.

[3] Bálint Tóth. Self-Interacting Random Motions. Progress in Mathematics, pages 555–564. Birkhäuser Basel, 2001.

[4] A. Ordemann, E. Tomer, G. Berkolaiko, S. Havlin, and A. Bunde. Structural properties of self-attracting walks. *Physical Review E*, 64(4):046117, September 2001.

[5] Jacob G. Foster, Peter Grassberger, and Maya Paczuski. Reinforced walks in two and three dimensions. *New Journal of Physics*, 11(2):023009, February 2009. Publisher: IOP Publishing.

[6] Angela Stevens and Hans G. Othmer. Aggregation, Blowup, and Collapse: The ABC's of Taxis in Reinforced Random Walks. *SIAM Journal on Applied Mathematics*, 57(4):1044–1081, August 1997.

[7] V. B. Sapozhnikov. Self-attracting walk with nu $\less$1/2. *Journal of Physics A: Mathematical and General*, 27(6):L151–L153, March 1994.

[8] Mihael Perman and Wendelin Werner. Perturbed Brownian motions. *Probability Theory and Related Fields*, 108(3):357–383, July 1997.

[9] Burgess Davis. Weak limits of perturbed random walks and the equation Y_t = B_t + \alpha\sup\{Y_s\colon s \leq t\}+\beta\inf\{Y\sb s\colon s \leq t\}. *Annals of Probability*, 24(4):2007–2023, October 1996. Publisher: Institute of Mathematical Statistics.

[10] M. A. Prasad, D. P. Bhatia, and D. Arora. Diffusive behaviour of self-attractive walks. *Journal of Physics A: Mathematical and General*, 29(12):3037–3040, June 1996.

[11] A. Barbier–Chebbah, O. Benichou, and R. Voituriez. Anomalous persistence exponents for normal yet aging diffusion. *Physical Review E*, 102(6):062115, December 2020. Publisher: American Physical Society.

[12] Alan J. Bray, Satya N. Majumdar, and G. Schehr. Persistence and First-Passage Properties in Non-equilibrium Systems. *Advances in Physics*, 62(3):225–361, June 2013. arXiv: 1304.1195.

[13] Peter Grassberger. Self-Trapping Self-Repelling Random Walks. *Physical Review Letters*, 119, August 2017.

[14] O. Bénichou, M. Coppey, M. Moreau, P. H. Suet, and R. Voituriez. Averaged residence times of stochastic motions in bounded domains. *EPL (Europhysics Letters)*, 70(1):42, March 2005. Publisher: IOP Publishing.
