# Supplementary material for: Cell migration guided by long-lived spatial memory
Source: Nat Commun. 2021 Jul 5;12:4118. doi: 10.1038/s41467-021-24249-8 (PMC8257581; doi:10.1038/s41467-021-24249-8)
Supplement: Supplementary file 3 — Description of Additional Supplementary Files [file 41467_2021_24249_MOESM3_ESM.pdf]

Description of Additional Supplementary Files

File Name: Supplementary Movie 1
Description: Oscillating MDCK cell on a 20 □um track. Phase contrast images. Scale bar 100 □um.

File Name: Supplementary Movie 2
Description: Static MDCK cell on a 20 □um track. Phase contrast images. Scale bar 100 □um.

File Name: Supplementary Movie 3
Description: Oscillating MDCK cell on a 20 □um track. Fluorescence intensity of PBD-YFP. Scale bar 50 □um.

File Name: Supplementary Movie 4
Description: MDCK cell on a 20 □um track, alternating between oscillatory and static phases. Fluorescence intensity of PBD-YFP. Scale bar 50 □um.

File Name: Supplementary Movie 5
Description: MDCK cell on a control (top, same video as Supplementary Video 1) and a conditionned (bottom) 20 □um track. Scale bars 100 □um.
