# Supplementary material for: Cell migration guided by long-lived spatial memory
Source: Nat Commun. 2021 Jul 5;12:4118. doi: 10.1038/s41467-021-24249-8 (PMC8257581; doi:10.1038/s41467-021-24249-8)
Supplement: Supplementary file 9 — Reporting Summary [file 41467_2021_24249_MOESM9_ESM.pdf]

nature research

Corresponding author(s): Benoît Ladoux, Raphaël Voituriez, Joseph d'Alessandro

Reporting Summary

Nature Research wishes to improve the reproducibility of the work that we publish. This form provides structure for consistency and transparency in reporting. For further information on Nature Research policies, see Authors & Referees and the Editorial Policy Checklist.

Statistical parameters

When statistical analyses are reported, confirm that the following items are present in the relevant location (e.g. figure legend, table legend, main text, or Methods section).

n/a	Confirmed	
☐	☒	The exact sample size (*n*) for each experimental group/condition, given as a discrete number and unit of measurement
☐	☒	An indication of whether measurements were taken from distinct samples or whether the same sample was measured repeatedly
☐	☒	The statistical test(s) used AND whether they are one- or two-sided<br>*Only common tests should be described solely by name; describe more complex techniques in the Methods section.*
☒	☐	A description of all covariates tested
☐	☒	A description of any assumptions or corrections, such as tests of normality and adjustment for multiple comparisons
☐	☒	A full description of the statistics including central tendency (e.g. means) or other basic estimates (e.g. regression coefficient) AND variation (e.g. standard deviation) or associated estimates of uncertainty (e.g. confidence intervals)
☐	☒	For null hypothesis testing, the test statistic (e.g. *F*, *t*, *r*) with confidence intervals, effect sizes, degrees of freedom and *P* value noted<br>*Give P values as exact values whenever suitable.*
☒	☐	For Bayesian analysis, information on the choice of priors and Markov chain Monte Carlo settings
☒	☐	For hierarchical and complex designs, identification of the appropriate level for tests and full reporting of outcomes
☒	☐	Estimates of effect sizes (e.g. Cohen's *d*, Pearson's *r*), indicating how they were calculated
☐	☒	Clearly defined error bars<br>*State explicitly what error bars represent (e.g. SD, SE, CI)*

Our web collection on statistics for biologists may be useful.

Software and code

Policy information about availability of computer code

Data collection	Metamorph 7.10.1.161
Data analysis	Images and raw data were quantified, analysed and plotted in Matlab 2018b and FIJI (ImageJ1.53c), Figures and cartoons were organized and prepared in INKSCAPE 1.0.1 respectively.

For manuscripts utilizing custom algorithms or software that are central to the research but not yet described in published literature, software must be made available to editors/reviewers upon request. We strongly encourage code deposition in a community repository (e.g. GitHub). See the Nature Research guidelines for submitting code & software for further information.

Data

Policy information about availability of data

All manuscripts must include a data availability statement. This statement should provide the following information, where applicable:

- Accession codes, unique identifiers, or web links for publicly available datasets
- A list of figures that have associated raw data
- A description of any restrictions on data availability

Analysis tools are available for purposes of reproducing or extending the analysis and other raw data will be available on general request.

Field-specific reporting

Please select the best fit for your research. If you are not sure, read the appropriate sections before making your selection.

☒ Life sciences ☐ Behavioural & social sciences ☐ Ecological, evolutionary & environmental sciences

For a reference copy of the document with all sections, see nature.com/authors/policies/ReportingSummary-flat.pdf

Life sciences study design

All studies must disclose on these points even when the disclosure is negative.

Sample size	Throughout the study, averages over multiple cells recorded during several independent experiments were performed and the results were successfully reproduced. Sample sizes (n = number of cell trajectories from distinct cells) are indicated at the relevant location in manuscript. Sample size calculation was not performed but n>100 is empirically known to provide robust results on single cell motility quantifications (MSD...) and smooth distributions of the data.
Data exclusions	Cells dividing or detaching within the first 10h of the experiments were excluded as we were interested in the long-term trajectories of isolated single cells only. These criteria were established a priori and apply to all experiments throughout the paper.
Replication	All the experiments were successfully repeated with similar results (see the main and supplementary figures for more details). For example, the central observation of this article -- MDCK cells oscillating with growing amplitude on linear patterns -- was consistently observed in 100% of >15 independent experiments (including even more technical replicates within single experiments) performed over a >4 years time period.
Randomization	Randomization is not relevant to this study as samples were processed and allocated according to specific experimental conditions defined by the experimenter.
Blinding	Blinding was unnecessary as data were most largely processed automatically using pre-defined parameters.

Reporting for specific materials, systems and methods

Materials & experimental systems

n/a	Involved in the study
☒	☐ Unique biological materials
☐	☒ Antibodies
☐	☒ Eukaryotic cell lines
☒	☐ Palaeontology
☒	☐ Animals and other organisms
☒	☐ Human research participants

Methods

n/a	Involved in the study
☒	☐ ChIP-seq
☒	☐ Flow cytometry
☒	☐ MRI-based neuroimaging

Antibodies

Antibodies used	Primary Antibodies:<br>Mouse monoclonal [IST-9] to Fibronectin - ab6328 (Abcam)<br>Rabbit to laminin - L9393 (Sigma-Aldrich)<br><br>Secondary Antibodies:<br>Alexa 488, goat anti-mouse (Life Technologies A11001)<br>Alexa 568, goat anti-rabbit (Life Technologies A11011)
Validation	All these antibodies has previously been used in published articles which are referred in the text and on the manufacturers' webpages (see antibodies references)

Eukaryotic cell lines

Policy information about cell lines

Cell line source(s)	MDCK-WT was obtained from ATCC, MDCK-YFP-PBD cells were generated and gifted by Fernando Martin-Belmonte from Universidad Autonoma de Madrid. MDCK-H1-GFP were generated from our MDCK-WT line and provided by Sham Tlili from

	Matière et Système Complexe lab (Paris). Caco2-shNT cells were generated in the team based on Caco2-wt cells from ATCC.
Authentication	Authentication was not performed in this study
Mycoplasma contamination	Cell lines tested negatively for the Mycoplasma contamination.
Commonly misidentified lines (See ICLAC register)	No commonly misidentified cell lines were used.
